# A systematic assessment of how rootstock growth characteristics impact grafted tomato plant biomass, resource partitioning, yield, and fruit mineral composition

**DOI:** 10.3389/fpls.2022.948656

**Published:** 2022-12-15

**Authors:** Tian Gong, Jeffrey K. Brecht, Karen E. Koch, Samuel F. Hutton, Xin Zhao

**Affiliations:** ^1^Horticultural Sciences Department, University of Florida, Gainesville, FL, United States; ^2^Gulf Coast Research and Education Center, University of Florida, Wimauma, FL, United States

**Keywords:** rootstock-scion interaction, rootstock-scion synergy, generative rootstock, vegetative rootstock, rootstock vigor, beefsteak tomato, grape tomato

## Abstract

The appropriate selection of rootstock-scion combinations to improve yield and fully realize grafting benefits requires an in-depth understanding of rootstock-scion synergy. Toward this end, we grafted two determinate-type scions [grape tomato (‘BHN 1022') and beefsteak tomato (‘Skyway')] onto four rootstocks with different characteristics to examine plant growth, yield performance, biomass production, and fruit mineral nutrient composition. The study was conducted during two growing seasons (spring and fall plantings in Florida) under organic production in high tunnels with the non-grafted scions as controls. Rootstocks had previously been designated as either “generative” (‘Estamino') or “vegetative” (‘DR0141TX') by some commercial suppliers or had not been characterized [‘RST-04-106-T' and ‘SHIELD RZ F1 (61-802)']. Also, ‘Estamino', ‘DR0141TX', and ‘RST-04-106-T' had been described as more vigorous than ‘SHIELD RZ F1 (61-802)'. In both planting seasons (with low levels of soilborne disease pressure), the “vegetative” and “generative” rootstocks increased marketable and total fruit yields for both scions except for the beefsteak tomato grafted with the “vegetative” rootstock in fall planting. Positive effects of ‘RST-04-106-T' on fruit yield varied with scions and planting seasons, and were most manifested when grafted with the beefsteak tomato scion in fall planting. ‘SHIELD RZ F1 (61-802)' led to similar yields as the non-grafted controls except for grafting with the grape tomato scion in fall planting. For vegetative and fruit biomass, both the “vegetative” and “generative” rootstocks had positive impacts except for the beefsteak tomato in fall planting. For fruit mineral composition, the “vegetative” and “generative” rootstocks, both highly vigorous, consistently elevated fruit P, K, Ca, Zn, and Fe contents on a dry weight basis, whereas the other rootstocks did not. Overall, although the more vigorous rootstocks enhanced tomato plant productivity and fruit minerals, the evidence presented here does not support the suggestion that the so-called “vegetative” and “generative” rootstocks have different impacts on tomato scion yield, biomass production, or fruit mineral contents. More studies with different production systems and environmental conditions as well as contrasting scion genotypes are needed to further categorize the impacts of rootstocks with different vigor and other characteristics on plant biomass production and their implications on fruit yield development.

## Introduction

Tomato (*Solanum lycopersicum*) grafting has been widely conducted because appropriately selected rootstocks can protect tomato scions from soilborne diseases and root-knot nematodes (Suchoff et al., 2019; Frey et al., 2020a) as well as abiotic stress (Abdelmageed and Gruda, 2009; Kumar et al., 2015; Zhang et al., 2019; Bristow et al., 2021). Beneficial effects of rootstocks have also been reported for tomato plant growth, fruit yield, nitrogen (N) use efficiency and N uptake efficiency, and nutrient accumulation (Turhan et al., 2011; Djidonou et al., 2017, 2019). In addition, tomato grafting has been increasingly used as a cultural practice of the integrated pest management program for organic production systems. Still, further potential lies ahead for grafting to enhance the high-value production of tomato in high tunnel systems, in which tomato is one of the most commonly grown crops worldwide (Carey et al., 2009; Lamont, 2009; Janke et al., 2017; Frey et al., 2020b).

Rootstock effects on tomato yield under non-stressed conditions have been investigated previously using rootstocks of different genetic backgrounds (interspecific vs. intraspecific) or vigor [either defined by commercial suppliers or in the current literature based on features such as shoot dry weight (DW) (Martínez-Andújar et al., 2017), single leaf size (Albacete et al., 2009), or the combination of emergence, seedling biomass accumulation, and stem and leaf features (Hu et al., 2016)]. Given the large number of rootstocks with diverse characteristics as well as numerous scion cultivars with different fruit sizes available and growth habits, the wide range of rootstock-scion combinations may lead to mixed results regarding performance of grafted plants. Different production environments further complicate rootstock effects on tomato scion growth, yield, and other physiological attributes. For example, with small-fruited tomato types under organic production in greenhouse conditions, Albino et al. (2018) showed that interspecific hybrid rootstock cultivars (*S. lycopersicum* × *S. habrochaites*) could have positive or neutral effects on fruit yield. In contrast, the *S. lycopersicum* × *S. pimpinellifolium* rootstock was found to negatively affect the fruit number of cherry tomato in greenhouse production compared with the non-grafted control (Mauro et al., 2020). For the beefsteak tomato grown in a high tunnel production system, Lang et al. (2020) demonstrated that *S. lycopersicum* × *S. habrochaites* rootstocks consistently increased the marketable yield compared with the non-grafted control, and the increase appeared to be more related to fruit number than single fruit weight. According to another greenhouse experiment by Djidonou et al. (2017), certain interspecific rootstocks increased the beefsteak tomato marketable yield relative to the non-grafted control, and the yield enhancement was ascribed to the increase in fruit number or single fruit weight depending on the rootstock used. On the other hand, Fullana-Pericàs et al. (2018) observed a decrease in total yield of a tomato scion (95–120 g/fruit) as a result of grafting with interspecific rootstocks. As reported by Djidonou et al. (2020), the influence of interspecific rootstocks on marketable and total yields of tomato could be affected by production systems. Although it has been suggested that the low vigor tendency of rootstocks could lead to less vigorous growth of grafted tomato plants (Mauro et al., 2020), very few studies have systematically examined the impact of rootstock vigor characteristics on yield components of different types of tomato scions.

In addition to rootstock genetic background and vigor, some commercial suppliers have begun to use the terms “vegetative” and “generative” to describe the effects of specific rootstocks on tomato scions. Lopez-Marin et al. (2017) suggested that vigorous “vegetative” rootstocks were more suitable for large-fruited tomato cultivars grown in long cropping cycles, and that “generative” rootstocks were better for small-fruited cultivars grown in any cropping cycles or for large-fruited cultivars in short cropping cycles, as “generative” rootstocks put more energy into reproductive vs. vegetative tissues. However, there is a lack of research-based evidence to support such recommendations. Some commercial suppliers have classified ‘DR0141TX' and ‘Estamino' as “vegetative” and “generative” rootstocks, respectively. However, these two rootstocks have not been tested in tomato grafting trials until recently (Lang et al., 2020; Gong et al., 2022). More information is needed to characterize their potential effects on the yield performance of different scion types.

Previous research on tomato rootstocks has included their impacts on the growth and biomass of scions in addition to fruit production. For small-fruited tomato, Albino et al. (2018) reported that three out of four rootstocks increased the height of grafted plants, and that all rootstocks increased plant leaf number. However, Mauro et al. (2020) found that rootstocks with different genetic backgrounds differed in their effects on production of grafted plant biomass, vegetative biomass, fruit biomass, and harvest index [fruit biomass (DW)/aboveground plant biomass (DW)]. For large-fruited tomato, rootstocks can have different impacts on plant height, stem diameter, and plant biomass (DW) at crop termination under high-tunnel (Lang et al., 2020) or greenhouse production (Djidonou et al., 2017). However, plant biomass production was usually determined at the end of the growing season without considering biomass loss due to leaf senescence or pruning, thus compromising the evaluation of whole-season biomass production. A closer look at the plant biomass produced during the entire production cycle could reveal processes contributing to yield effects of different rootstocks as well as features of purported “vegetative” and “generative” rootstocks.

Although considerable attention has been directed to the influence of tomato rootstock genotype on overall mineral uptake and leaf nutrient content (Martínez-Ballesta et al., 2010; Singh et al., 2017), little research has assessed the mineral composition of tomato fruits. Kumar et al. (2015) reported that *S. lycopersicum* × *S. habrochaites* rootstocks increased fruit N and iron (Fe) but did not affect the contents of phosphorus (P), potassium (K), calcium (Ca), magnesium (Mg), zinc (Zn), manganese (Mn), and copper (Cu) on the dry weight basis of a medium-sized tomato (around 90 g/fruit) regardless of nickel (Ni) stress. It has also been suggested that the efficiency in absorbing and transporting certain minerals to tomato scions may vary with rootstock types (Goto et al., 2013), which could potentially impact fruit mineral availability in different grafting combinations.

In this study, four rootstocks were selected based on phenotypic analysis of their vigor and other growth characteristics (Gong, 2022). ‘DR0141TX' and ‘Estamino' (*S. lycopersicum* × *S. habrochaites*) were classified as vigorous, ‘SHIELD RZ F1 (61-802)' (*S. lycopersicum*) as low vigor, and ‘RST-04-106-T' as intermediate. The scions used were either the large-fruited ‘Skyway' beefsteak tomato or the small-fruited ‘BHN 1022' grape tomato, and the plants were grown in an organically managed high tunnel system during two production seasons. The objectives were to: (1) determine the effects of rootstocks with different vigor, genetic backgrounds, and other characteristics (vegetative vs. generative) on tomato scion growth, yield, and biomass production as well as fruit mineral contents, and (2) compare the responses of the beefsteak tomato and grape tomato scions to grafting.

## Materials and methods

### Experimental material

This study was conducted in the spring (hereafter referred to as spring planting) and fall (hereafter referred to as fall planting) production seasons in Florida from 2020 to 2021. In both planting seasons, the determinate ‘BHN 1022' grape tomato (BNHSeed, Immokalee, FL, United States) and the ‘Skyway' beefsteak tomato (Johnny's Selected Seeds, Winslow, ME, United States) were grafted onto the following tomato rootstocks: ‘DR0141TX' (vegetative) (De Ruiter Seeds, Bergschenhoek, Netherlands), ‘Estamino' (generative) (Vitalis Organic Seed, Salinas, CA, United States), ‘RST-04-106-T' (uncharacterized) (NE Seed, East Hartford, CT, United States), and ‘SHIELD RZ F1 (61-802)' (hereafter referred to as ‘Shield', uncharacterized) (Rijk Zwaan, De Lier, the Netherlands).

The tomato rootstocks and scions were seeded on 24 and 28 December 2019, respectively, for spring planting and on 1 and 5 August 2020, respectively, for fall planting in the greenhouse. Seeds were sown in 72-cell Speedling trays (Speedling Inc., Ruskin, FL, United States) filled with PROMIX premium organic vegetable and herb mix (Premier Tech Horticulture, Quakertown, PA, United States). Grafting was conducted when the plants had three to four true leaves, and on 21 January and 7 September 2020 for the spring and fall plantings, respectively (0 day after grafting, DAG). The splice grafting method (Lee and Oda, 2002) was used, and the plants were cut below the cotyledons of the rootstocks and between the cotyledon and the first true leaf of the scions.

### Setup of the high tunnel grafted tomato experiments

The high tunnel tomato production experiments were conducted on certified organic land at the University of Florida Plant Science Research and Education Unit (PSREU) in Citra, FL. A split-plot design with four replications was used with eight plants per subplot. One polyethylene film (0.152 mm)-covered caterpillar high tunnel (2.76 m high, 4.27 m wide, and 30.48 m long; Farmers Friends, Williamsport, TN, United States) served as a replication. Four north-south-oriented caterpillar high tunnels were spaced 3.05 m apart. Scion type (beefsteak and grape tomatoes) was the whole plot factor, and the non-grafted scion controls and grafting treatments with different rootstocks were randomized in the subplots.

The soil consisted of 95.1% sand, 1.3% clay, and 3.6% silt with 0.6% organic matter. Two raised beds were made with a between-bed (center to center) spacing of 1.83 m. Plants of the spring and fall plantings were transplanted on 14 February and 24 September 2020, respectively, into raised beds covered by black plastic mulch. For the spring planting, week 0 referred to the period of 14–15 February, week 1 referred to the period of 16–22 February, and so forth. For the fall planting, week 0 referred to the period of 25–26 September, week 1 referred to the period of 27 September–3 October, and so forth. The planting beds were 15 cm high and 63 cm wide in the spring and 10 cm high and 76 cm wide in the fall, with a 0.61 m plant spacing within the bed. The buffer zone between each subplot was 0.61 m, and the buffer zone between the different scion types was 1.83 m. The 1.83 m-wide buffer zones were also included in the front and back of each bed. Single-line drip tape (15 cm emitter spacing) was used with a flow rate at 1.9 L/min per 30.5 m (Jain Irrigation Systems Ltd., Bambhori, India).

Yard waste compost (Watson C&D, Gainesville, FL, United States) was applied to the planting beds at 22.4 t/ha in the spring and 16.8 t/ha in the fall. For each production season, preplant organic fertilizer 10-2-8 (Nature Safe; Darling Ingredients Inc., Irving, TX, United States) was applied to each raised bed at an N rate of 112 kg/ha. In-season fertigation was provided by applying weekly injections of a 5-1-1 liquid fish fertilizer (Aqua Power 5-1-1; JH Biotech, Inc., Ventura, CA, United States) and 0-0-50 potassium sulfate (Big K; JH Biotech, Inc.) at 11.8–34.0 kg/ha for N and 9.8–28.2 kg/ha for K, starting 2 weeks after transplanting (WAT) in both seasons. MgSO_4_ (Epsom salt) (Valudor Products, LLC, Encinitas, CA, United States) was injected at a rate of 11.2–17.9 or 12.0–19.0 kg/ha for spring planting (on 2, 9, and 16 March and 4 May) and fall planting (on 26 October, 9 November, and 21 December 2020 and 4, 11, 18, 25 January and 1 and 8 February 2021), respectively. In the fall planting, supplemental Ca (Biomin^®^ Calcium; JHBiotech, Inc.) was foliar-sprayed at 4.7 L/ha on 12 March 2021.

At 3 WAT, the plants were staked and stringed using the Florida weave method. Strings were added about every 10 or 14 days for the grape tomato and the beefsteak tomato, respectively, until late harvest. Sweet alyssum (*Lobularia maritima*) was seeded as an intercrop (at the bed ends and in the middle buffer zone of each bed) on 31 January and 24 September 2020 for the spring and fall plantings, respectively, to help with on-site enhancement of biodiversity for biological control. After the spring production season, cowpea (*Vigna unguiculata*) as a rotational cover crop was seeded on 9 July at a rate of 112 kg/ha and terminated on 24 August.

During cold months with frost events or near-freezing temperatures, both sidewalls of each high tunnel were closed to prevent chilling injury. One to two layers of row cover (PRO 50; AgriFabric, Spartanburg, SC, United States) was put on top of the high tunnel film when needed. In the spring planting, a 4.0 × 30.5 m 30% shading cloth (Svensson, Charlotte, NC, United States) was put on the top of each high tunnel on 1 May 2020 to reduce heat stress.

**Figure 1:**
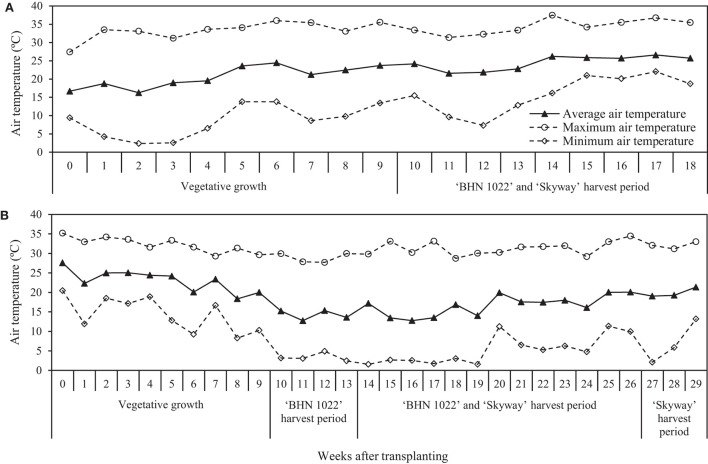
Weekly average, maximum, and minimum air temperatures at 1 m above the planting bed in the center of the high tunnel in **(A)** spring planting (data collected from 15 February to 18 June 2020) and **(B)** fall planting (data collected from 25 September 2020 to 15 April 2021). For the spring planting, week 0 included 15 February, week 1 included the period of 16–22 February, and so forth. For the fall planting, week 0 included the period of 25–26 September, week 1 included the period of 27 September–3 October, and so forth.

During the tomato production season, air temperature and relative humidity (RH) at 1 m above the planting bed in the middle of the high tunnel were recorded every 15 min using HOBO data loggers (MX2305; Onset Corp., Bourne, MA, United States) within a solar radiation shield (Onset Corp.). The whole season average, maximum, and minimum air tempdratures were 22.7, 37.5, and 2.4°C for the spring planting and 18.6, 35.2, and 1.5°C for the fall planting. The weekly average, maximum, and minimum temperatures for the spring and fall plantings are shown in Figure 1.

### Fruit yield components

Tomato harvests began on 20 April 2020 (10 WAT) for both the beefsteak tomato and the grape tomato in the spring planting. In the fall planting, the first harvest of the grape tomato was on 29 November 2020 (10 WAT), while the harvest of the beefsteak tomato took place on 28 December 2020 (14 WAT). Fruits were harvested twice per week until the end of the season. Grape tomatoes were harvested when they reached a uniform, complete red color with a tinge of orange, while beefsteak tomatoes were harvested when they reached at least the breaker stage [definite break in tan, pink, or red color; up to 10% of surface (Sargent, 1997)]. Harvested fruit were classified as either marketable or unmarketable, weighed, and counted. Undersize fruit (weighing <5 g for grape tomato or <100 g for beefsteak tomato) or fruit with more than 30% stink bug damage over the surface, cracking, damage by other pests, or showing any disease symptoms were classified as unmarketable. Otherwise, the fruit were classified as marketable. The harvesting ceased on 11 June 2020 for both the grape and beefsteak tomato cultivars in the spring planting, and on 31 March 2021 for the grape tomato and 8 April 2021 for the beefsteak tomato in the fall planting. At the final harvest, all fruit longer than 1 cm were harvested for both the beefsteak tomato and the grape tomato. The fruit were subsequently classified as green (fruit that did not reach breaker stage) or fruit in breaker or more advanced ripeness stages. Because green fruit are also an indication of productivity, it is reasonable to include them for understanding the total yield potential. Fruit within each group were further categorized as marketable or unmarketable based on their weight and presence of defects or pest/disease damage.

In order to better understand the impacts of the rootstocks on fruit yield components, the cumulative yield at the end of each week was calculated. The green fruits at the final harvest were excluded as they did not meet the harvest criteria based on fruit ripeness. Average marketable fruit weight was calculated by dividing whole-season marketable yield by whole-season marketable fruit number.

### Flower and fruit cluster counting

In the fall planting, during crop production and at crop termination, inflorescences, flower clusters, and fruit clusters were counted on two plants per subplot of the non-grafted and grafted ‘BHN 1022' grape tomato treatments. This was conducted on 18 November 2020 (55 DAT), 5 January 2021 (103 DAT), and 30 March 2021(191 DAT). An inflorescence was arbitrarily defined as being longer than 2 cm immature flower cluster with no open flowers. A flower cluster was defined as with at least one open flower and with no fruit longer than 1 cm. A fruit cluster was defined as having at least one fruit ≥1 cm in length.

### Plant growth measurement and destructive sampling after the final harvest

Plant height and stem diameter were measured at 18, 32, 63, 86, 101, and 119 DAT in the spring planting and 26, 42, 62, 81, 127, and 179 DAT in the fall planting on three plants in each subplot. Plant height was measured from the ground to the tip of the highest branch (Fullana-Pericàs et al., 2018), and stem diameter was measured at 5 cm above the ground (about 3 cm above the graft union).

Destructive sampling of both scions in the spring planting was conducted from 15 to 18 June 2020 following the final harvest. In the fall planting, destructive sampling of the grape tomato plants was conducted from 30 March to 5 April 2021, and from 8 to 15 April 2021 for the beefsteak tomato. In the spring planting, four plants per subplot were sampled, while in the fall planting, two plants per subplot were sampled. Because of the workload, plant sampling was conducted over consecutive days, and plants from the same block were sampled within the same day.

For the destructive sampling, plants were cut at ground level. The reproductive tissue (flower clusters and all fruit shorter than 1 cm at final harvest as well as any newly developed fruit) and vegetative tissue (leaves and stems) were separated, and each was dried at 65°C until constant weight for dry weight determination.

### Biomass collection throughout the production season and aboveground dry weight estimation

In both planting seasons, leaves that were touching the ground, senescent, or severely affected by pests or diseases were pruned and collected from each subplot every 2 weeks starting 6 WAT until 14 and 24 WAT for the spring and fall plantings, respectively. All the pruned leaves were dried at 65°C until constant weight for dry biomass determination. Whole-season vegetative biomass accumulation (dry weight based) was calculated as the sum of pruned biomass and vegetative biomass at destructive sampling on a per plant basis.

Whole-season plant reproductive biomass was calculated as the sum of harvested fruit dry biomass and the reproductive tissue dry weight at destructive sampling. Harvested fruit dry biomass was estimated by multiplying fresh fruit yield by corresponding fruit dry matter content. According to Abou Aziz (1968) and based on our previous studies (data not shown), tomato fruit dry matter content varied with ripeness stage. In addition, dry matter content also differs between grape and beefsteak tomatoes. Therefore, green fruit and fruit reaching breaker or more advanced stages for each scion were measured separately for dry matter content.

Red fruit of ‘BHN 1022' were sampled on 14 May 2020 (spring planting) and 8 January 2021 (fall planting) for measurement of dry matter content. The sampling of ‘Skyway' fruits took place on 21 May 2020 (spring planting) and 2 February 2021 (fall planting), while breaker-stage fruits were ripened at ambient temperature until the red ripe stage before dry matter content measurement. Approximately 600 g of ‘BHN 1022' and ‘Skyway' fruits from each subplot were homogenized using 908™ Commercial Bar Blender (HBB908; Hamilton Beach Brands, Inc., Glen Allen, VA, United States) under yellow light. About 100 g of each homogenized sample was poured into an aluminum bowl and dried at 65°C until constant weight. The dry matter content of tomato fruit was calculated as the ratio of dry weight to fresh weight and expressed in percentage.

A similar approach was used for assessing dry matter content for green fruits. However, because of the COVID pandemic, green fruit dry matter content was only measured in the fall planting. Moreover, green fruit across all the treatment plots were pooled to determine the dry matter content for each scion at final harvest assuming a little variation among the treatments. At the final harvest, four batches (each containing about 150 g green grape tomato fruit or about 700 g green beefsteak tomato fruit) were sampled from the pool, and the average of the four batches was used to represent the dry matter content of green fruits of each scion.

The aboveground biomass (hereafter referred to as plant biomass) was calculated as the sum of vegetative biomass and reproductive biomass. Harvest index was calculated as the ratio of reproductive biomass to plant biomass (Mauro et al., 2020).

### Tomato fruit mineral status at peak harvest

After measuring the dry matter content, dried fruit samples from peak harvest in both plantings were sent to Waters Agricultural Laboratories (Camilla, GA, United States) to measure the contents of macronutrients, including N, P, sulfur (S), K, Ca, and Mg, and micronutrients including boron (B), Zn, Mn, Fe, and Cu. Fruit mineral contents were reported on a dry weight basis.

### Assessment of root-knot nematode infestation

When the destructive sampling was conducted, root-knot nematode (RKN) infestation on plant roots was also assessed, as root galling in some plants was visible. Basically, roots of each plant from the top 30 cm of the soil and within 30 cm from the main stem were dug up, and soil particles were gently removed (Gong et al., 2022). All plants in each subplot were assessed for nematode galls using a 0–10 rating scale (Zeck, 1971): 0 = no galling, 10 = plant and roots are dead. Two researchers assessed each plant individually, and ratings were averaged for each subplot (Barrett et al., 2012).

### Statistical analyses

Data from the two planting seasons were analyzed separately because of substantial differences in growth, yield, and biomass produced during the two seasons. Whole-season yields, biomass accumulation, and fruit mineral content were analyzed following a split-plot design using a generalized linear mixed model in the GLIMMIX procedure of SAS (version 9.4; SAS Institute, Cary, NC, United States). Because of the distinct growth habit of grape tomato and beefsteak tomato, plant height and stem diameter in different growth stages were analyzed using repeated measures. Cumulative yields of each harvest week of the two scion cultivars were analyzed separately using a randomized complete block design to compare the non-grafted and grafted plant treatments. Numbers of inflorescences, flower clusters, and fruit clusters of grape tomato treatments in the fall planting were also analyzed using a randomized complete block design. Square root transformation was conducted for some data of yield components, fruit mineral contents, plant height, inflorescence, flower cluster, and fruit cluster as needed to meet the model assumptions, and the results were presented using the original data. Fisher's least significant difference (LSD) test at *P* ≤ 0.05 was conducted for multiple comparisons of different measurements among treatments. In addition, a Pearson correlation analysis was conducted using SAS (version 9.4; SAS Institute) to determine the relations between the parameters of biomass production, partitioning, and total yield. The RKN galling index ratings of each scion in each planting season were analyzed following a randomized complete block design by non-parametric analysis in JMP Pro 15 (SAS Institute) based on the Wilcoxon method. A Wilcoxon Each Pair test at *P* ≤ 0.05 was conducted for multiple comparisons among the treatments.

## Results

### Plant height and stem diameter

**Figure 2:**
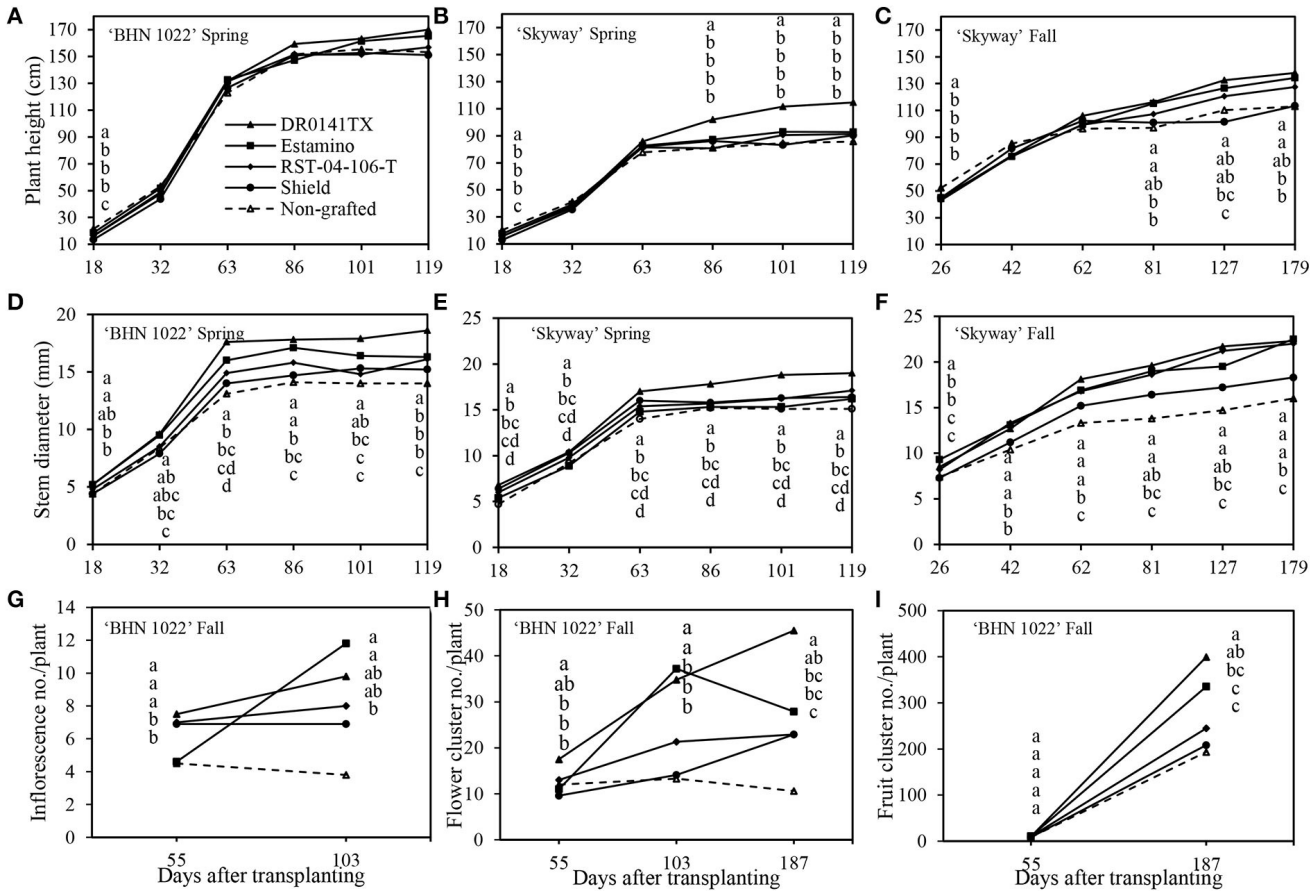
Impacts of the four rootstocks on plant height, stem diameter, and floral features in the spring (14 February to 18 June 2020) and fall (24 September 2020 to 15 April 2021) plantings. Vegetative features are compared for the ‘BHN 1022' grape tomato and ‘Skyway' beefsteak tomato scions grafted onto different rootstocks. The inflorescence number, flower cluster number, and fruit cluster number were assessed for ‘BHN 1022' grape tomato scion grafted with different rootstocks at 55 (before the 1st harvest of grape tomato), 103 (harvest week 6), and 187 DAT (at crop termination) in the fall planting. **(A)** Plant height of ‘BHN 1022' in the spring planting. **(B)** Plant height of ‘Skyway' in the spring planting. **(C)** Plant height of ‘Skyway' in the fall planting. **(D)** Stem diameter of ‘BHN 1022' in the spring planting. **(E)** Stem diameter of ‘Skyway' in the spring planting. **(F)** Stem diameter of ‘Skyway' in the fall planting. **(G)** Inflorescence number of ‘BHN 1022' in the fall planting. **(H)** Flower cluster number of ‘BHN 1022' in the fall planting. **(I)** Fruit cluster number of ‘BHN 1022' in the fall planting. For the same measuring date, data with the same letter are not significantly different at *P* ≤ 0.05 according to Fisher's LSD test.

The profiles of plant height and stem diameter over time showed different trajectories. Initially, all the plants grew rapidly regardless of grafting status or planting season. For the ‘BHN 1022' grape tomato in the spring planting, all the rootstocks decreased plant height relative to the non-grafted control at 18 DAT, but after that, no differences in rootstock effects were detected (Figure 2A). The rootstock effect on the plant height of ‘BHN 1022' in the fall planting showed a different pattern, which was not influenced by plant growth stage (data not shown). Overall, ‘DR0141TX' and ‘Estamino' increased plant height by 5.3% compared with the non-grafted control, which did not differ from the ‘RST-04-106-T' and ‘Shield' treatments. In addition, ‘BHN 1022' grafted onto ‘DR0141TX', ‘Estamino', or ‘RST-04-106-T' was also taller than ‘Shield'. For the large-fruited ‘Skyway' scion, the rootstock impacts on plant height varied with plant stage in both the spring and fall plantings. At the first measuring date of each season, the non-grafted ‘Skyway' was taller than all the other grafting treatments (Figures 2B,C), but differences disappeared at the next two growth stages. In the spring planting, plants grafted with ‘DR0141TX' were taller than all the other treatments at 86, 101, and 119 DAT, while the remaining treatments were similar. In the fall planting, plants grafted onto ‘DR0141TX' or ‘Estamino' were taller than the non-grafted control at 81, 127, and 179 DAT. Plants grafted with ‘RST-04-106-T' were also taller than the non-grafted control at 127 DAT.

In the spring planting, the rootstock effect on the stem diameter of ‘BHN 1022' differed among plant growth stages. At 18 DAT, the vigorous rootstocks (‘DR0141TX' and ‘Estamino') led to thicker stems than the less vigorous ‘Shield' or the non-grafted control (Figure 2D). Later (from 63 DAT until 119 DAT), scion plants grafted onto ‘DR0141TX', ‘Estamino', or ‘RST-04-106-T' generally had greater stem diameters than the non-grafted control (except at 101 DAT). Only at 119 DAT did scion plants grafted onto ‘Shield' have a thicker stem than the non-grafted control. In addition, at 119 DAT, ‘DR0141TX' had the greatest stem diameter among all the rootstock treatments. In the fall planting, similar rootstock impacts on the stem diameter of ‘BHN 1022' were observed across plant growth stages (data not shown). ‘DR0141TX', ‘Estamino', or ‘RST-04-106-T' rootstocks increased the stem diameter by 13.6% compared with the non-grafted ‘BHN 1022', while no difference was found between the ‘Shield' treatment and the non-grafted control. In the spring, grafting ‘Skyway' with ‘DR0141TX', ‘Estamino', or ‘RST-04-106-T' resulted in greater stem diameter compared with the non-grafted control across all the measuring dates. Plants grafted with ‘Shield' did not differ from those grafted with ‘RST-04-106-T' and the non-grafted control (Figure 2E). Furthermore, ‘DR0141TX' also had greater stem diameter than ‘Estamino' and ‘RST-04-106-T', while the latter two were similar. In the fall planting, ‘Skyway' grafted onto ‘DR0141TX', ‘Estamino', or ‘RST-04-106-T' had thicker stems than the non-grafted control across all the measuring dates, whereas the stem diameter increase by ‘Shield' was only observed at 62 and 179 DAT (Figure 2F).

### Fruit yield components

Both the rootstocks and the scions showed main effects on whole-season marketable and total fruit yields in the spring planting, and their interactions also became evident in the fall planting. In the spring planting, grafting with the vigorous rootstocks (‘DR0141TX' and ‘Estamino') increased the marketable yield by 29.4% compared with the other treatments for both scions, among which no differences were detected (Table 1). Scions grafted onto ‘DR0141TX' produced the greatest total yield, followed by those on ‘Estamino', and then by ‘RST-04-106-T', which were respectively 54.5, 38.6, and 13.6% higher than the non-grafted controls. The least vigorous rootstock ‘Shield' did not impact total fruit yield under these conditions. In the spring planting, large-fruited ‘Skyway' scions produced greater marketable and total fruit yields than the small-fruited ‘BHN 1022' (Table 1). In the fall planting, all the four rootstocks increased the marketable yield of the ‘BHN 1022' scion by an average of 82.5% relative to the non-grafted control (Table 2). Grafting with ‘DR0141TX' and ‘Estamino' led to 103.8 and 122.6% greater marketable yield than the non-grafted control, and these two rootstocks also resulted in 44.0 and 57.3%, respectively, greater marketable yield than did the less vigorous ‘Shield'. The rootstocks increased the total yield of the ‘BHN 1022' scion by an average of 79.2% relative to the non-grafted control, with the vigorous ‘DR0141TX' and ‘Estamino' increasing the yield by an average of 43.1% more than the other rootstocks (‘RST-04-106-T' and ‘Shield'). For the large-fruited ‘Skyway' scion, both the vigorous ‘Estamino' and the medium-vigorous ‘RST-04-106-T' rootstocks increased the marketable and total yields by averages of 44.7 and 34.0%, respectively, compared with the least vigorous ‘Shield' and the non-grafted control. The results for grafting ‘Skyway' onto the vigorous ‘DR0141TX' rootstock were similar to those of the other rootstocks. In addition, the large-fruited ‘Skyway' produced greater marketable and total yields than the small-fruited grape tomato ‘BHN 1022' when it was grafted with ‘RST-04-106-T' or remained ungrafted. Intrinsic differences in growth habits of the grape tomato and the beefsteak tomato emerged most prominently in rootstock-scion interaction effects for the numbers of total and marketable fruit produced per plant during both planting seasons. In the spring, the ‘BHN 1022' grape tomato grafted onto the ‘DR0141TX', ‘Estamino', or ‘RST-04-106-T' rootstock produced an average of 31.5 and 23.7% more total and marketable fruit per plant, respectively, than the ‘Shield' treatment and the non-grafted control (Table 1). The ‘BHN 1022' plants grafted with the vigorous ‘DR0141TX' rootstock produced a greater number of marketable and total fruit than the medium-vigorous ‘RST-04-106-T' treatment. In addition, grafting onto ‘DR0141TX' also led to greater total fruit number than ‘Estamino'. In the fall, all the rootstocks increased the marketable fruit number of ‘BHN 1022' by an average of 65.0% compared with the non-grafted control (Table 2). The vigorous rootstocks (‘DR0141TX' and ‘Estamino') produced similar amounts of marketable grape tomato fruits, which were 39.0% greater than the average of the ‘RST-04-106-T' and ‘Shield' treatments. A similar trend was also observed for the total fruit number of ‘BHN 1022'. For both spring and fall plantings, the marketable and total fruit numbers of the ‘Skyway' beefsteak tomato scion were not significantly affected by the rootstocks (Tables 1, 2).

**Table 1:** Marketable and total fruit yields and numbers of fruit per plant of the grafted ‘BHN 1022' grape tomato and ‘Skyway' beefsteak tomato scions in the spring planting (14 February to 18 June 2020).

**Treatment**	**Marketable fruit yield (kg/plant)**	**Total fruit yield (kg/plant)**	**Marketable fruit number** **(no./plant)**		**Total fruit number** **(no./plant)**	
			**BHN 1022**	**Skyway**	**BHN 1022**	**Skyway**
**Rootstock (Rs)**						
DR0141TX	4.6 ± 0.4a	6.8 ± 0.7a	363.7 ± 19.4Aa	17.0 ± 4.6Ba	515.9 ± 30.9Aa	37.6 ± 9.0Ba
Estamino	4.8 ± 0.4a	6.1 ± 0.7b	341.2 ± 18.8Aab	18.7 ± 4.8Ba	449.0 ± 28.9Ab	32.7 ± 8.5Ba
RST-04-106-T	3.9 ± 0.4b	5.0 ± 0.6c	325.7 ± 18.4Ab	13.8 ± 4.2Ba	428.4 ± 28.2Ab	28.2 ± 7.9Ba
Shield	3.5 ± 0.4b	4.3 ± 0.6d	286.0 ± 17.2Ac	13.8 ± 4.2Ba	357.5 ± 25.9Ac	25.9 ± 7.6Ba
Non-grafted	3.5 ± 0.4b	4.4 ± 0.6d	269.3 ± 16.7Ac	14.0 ± 4.2Ba	348.9 ± 25.6Ac	29.5 ± 8.1Ba
**Scion (Sc)**						
BHN 1022	3.3 ± 0.4b	3.9 ± 0.6b	–		–	
Skyway	4.8 ± 0.4a	6.8 ± 0.8a	–		–	
***P*** **-Value**						
Rs	<0.001	<0.001	<0.001		<0.001	
Sc	<0.001	<0.001	<0.001		<0.001	
Rs × Sc	0.227	0.127	0.011		<0.001	

DR0141TX, tomato scions grafted onto rootstock ‘DR0141TX'; Estamino, tomato scions grafted onto rootstock ‘Estamino'; RST-04-106-T, tomato scions grafted onto rootstock ‘RST-04-106-T'; Shield, tomato scions grafted onto ‘Shield'; Non-grafted, non-grafted tomato scion controls.

Mean ± SE (standard error); means followed by the same uppercase letter within a row, and means followed by the same lowercase letter within a column are not significantly different at P ≤ 0.05 according to Fisher's LSD test.

**Table 2:** Marketable and total fruit yields and numbers of fruit per plant from the grafted ‘BHN 1022' grape tomato and ‘Skyway' beefsteak tomato scions in the fall planting (24 September 2020 to 15 April 2021).

**Treatment**	**Marketable fruit yield (kg/plant)**		**Total fruit yield (kg/plant)**		**Marketable fruit number (no./plant)**		**Total fruit number (no./plant)**	
	**BHN 1022**	**Skyway**	**BHN 1022**	**Skyway**	**BHN 1022**	**Skyway**	**BHN 1022**	**Skyway**
DR0141TX	10.8 ± 1.6Aab	10.8 ± 1.6Aab	12.0 ± 1.8Aa	14.1 ± 1.9Aab	1,123.3 ± 88.7Aa	60.8 ± 23.2Ba	1,437.5 ± 106.3Aa	110.3 ± 32.1Ba
Estamino	11.8 ± 1.7Aa	12.3 ± 1.7Aa	12.9 ± 1.9Aa	16.2 ± 2.1Aa	1,210.7 ± 92.0Aa	70.7 ± 24.7Ba	1,557.7 ± 110.5Aa	132.8 ± 34.9Ba
RST-04-106-T	8.6 ± 1.5Bbc	13.6 ± 1.8Aa	9.3 ± 1.6Bb	16.9 ± 2.1Aa	893.8 ± 79.5Ab	75.0 ± 25.4Ba	1,114.2 ± 94.0Ab	126.0 ± 34.0Ba
Shield	7.5 ± 1.4Ac	9.3 ± 1.5Ab	8.1 ± 1.5Bb	12.6 ± 1.8Ab	785.0 ± 74.7Ab	55.7 ± 22.3Ba	962.9 ± 87.7Abc	107.8 ± 31.8Ba
Non-grafted	5.3 ± 1.2Bd	8.6 ± 1.5Ab	5.9 ± 1.3Bc	12.1 ± 1.8Ab	607.9 ± 66.1Ac	53.2 ± 21.9Ba	785.2 ± 79.5Ac	108.7 ± 31.9Ba
***P*** **-Value**								
Rs	<0.001		<0.001		<0.001		<0.001	
Sc	0.070		0.020		<0.001		<0.001	
Rs × Sc	0.040		0.030		<0.001		<0.001	

DR0141TX, tomato scions grafted onto rootstock ‘DR0141TX'; Estamino, tomato scions grafted onto rootstock ‘Estamino'; RST-04-106-T, tomato scions grafted onto rootstock ‘RST-04-106-T'; Shield, tomato scions grafted onto ‘Shield'; Non-grafted, non-grafted tomato scion controls.

Mean ± SE (standard error); means followed by the same uppercase letter within a row and means followed by the same lowercase letter within a column are not significantly different at P ≤ 0.05 according to Fisher's LSD test.

Rs, rootstock; Sc, scion; Rs × Sc, rootstock × scion interaction.

Although average marketable fruit weight did not differ among the rootstocks in the spring for either scion, differences emerged in the fall (Table 3). In the fall planting, the ‘DR0141TX', ‘Estamino', and ‘RST-04-106-T' rootstocks increased the average marketable fruit weight by 10.8, 9.2, and 12.0%, respectively, compared with the non-grafted controls. The effects of the ‘Shield' rootstock were minimal.

**Table 3:** Average marketable fruit weight from the grafted ‘BHN 1022' grape tomato and ‘Skyway' beefsteak tomato scions in the spring planting (14 February to 18 June 2020) and the fall planting (24 September 2020 to 15 April 2021).

**Treatment**	**Spring planting**	**Fall planting**
**Rootstock (Rs)**
DR0141TX	111.4 ± 4.3	67.6 ± 2.0ab
Estamino	113.4 ± 4.4	66.6 ± 2.0ab
RST-04-106	110.5 ± 4.3	68.3 ± 2.0a
Shield	105.3 ± 4.2	63.8 ± 1.9bc
Non-Grafted	108.2 ± 4.3	61.0 ± 1.9c
**Scion (Sc)**
BHN 1022	10.4 ± 1.4b	9.4 ± 0.8b
Skyway	314.3 ± 7.0a	171.8 ± 3.1a
***P*** **-Value**
Rs	0.531	0.010
Sc	<0.001	<0.001
Rs × Sc	0.774	0.132

DR0141TX, tomato scions grafted onto rootstock ‘DR0141TX'; Estamino, tomato scions grafted onto rootstock ‘Estamino'; RST-04-106-T, tomato scions grafted onto rootstock ‘RST-04-106-T'; Shield, tomato scions grafted onto ‘Shield'; Non-grafted, non-grafted tomato scion controls.

Mean ± SE (standard error); means within a column followed by the same letter are not significantly different at P ≤ 0.05 according to Fisher's LSD test.

### Cumulative yield during growing seasons

**Figure 3:**
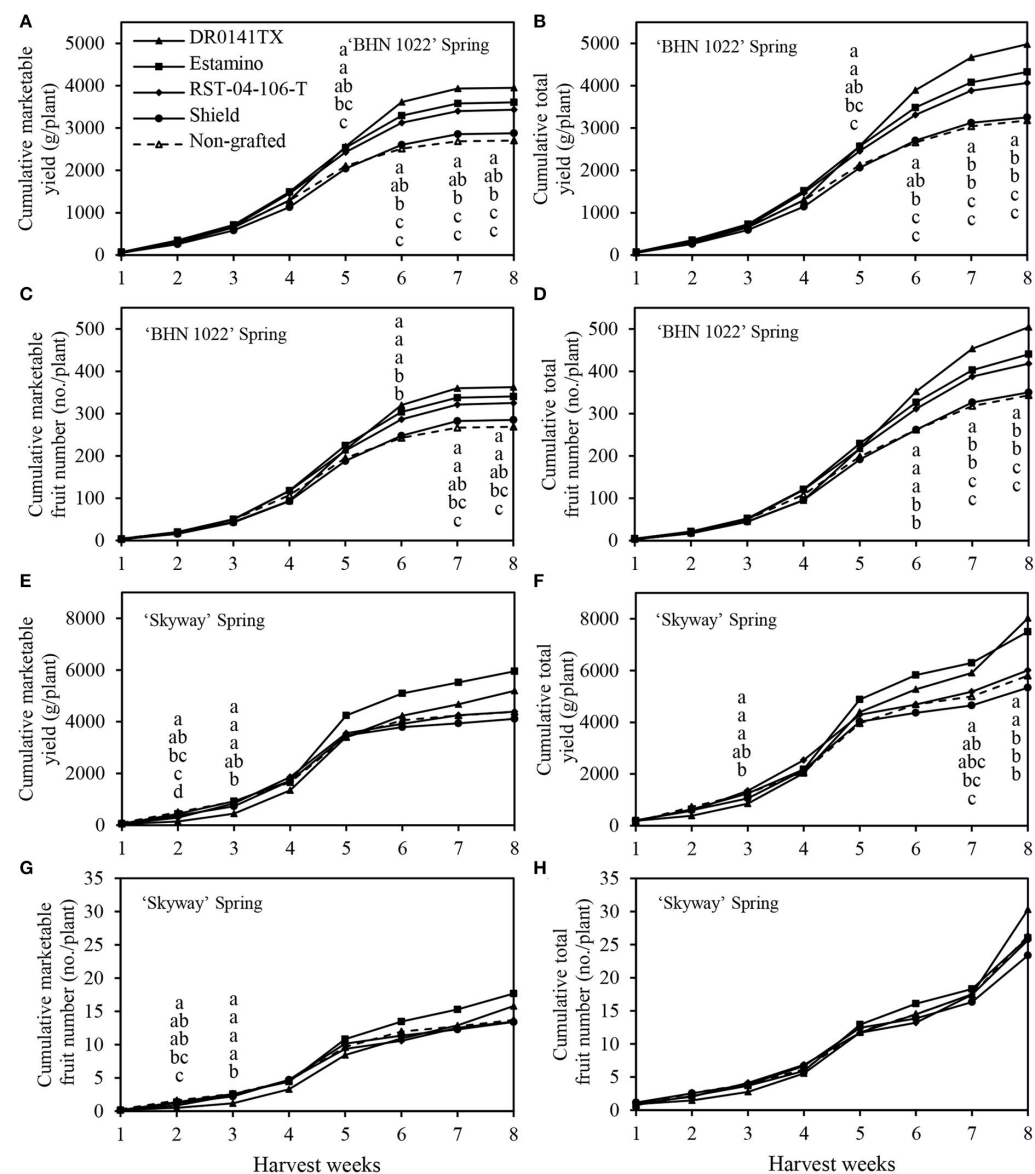
Cumulative marketable and total fruit yields and numbers per plant of the grafted ‘BHN 1022' grape tomato and ‘Skyway' beefsteak tomato scions during the spring growing season (14 February to 18 June 2020). Green fruits at final harvest were excluded. **(A)** Cumulative marketable yield of ‘BHN 1022'. **(B)** Cumulative total yield of ‘BHN 1022'. **(C)** Cumulative marketable fruit number of ‘BHN 1022'. **(D)** Cumulative total fruit number of ‘BHN 1022'. **(E)** Cumulative marketable yield of ‘Skyway'. **(F)** Cumulative total yield of ‘Skyway'. **(G)** Cumulative marketable fruit number of ‘Skyway'. **(H)** Cumulative total fruit number of ‘Skyway'. For the same harvest week, data with the same letter are not significantly different at *P* ≤ 0.05 according to Fisher's LSD test.

**Figure 4:**
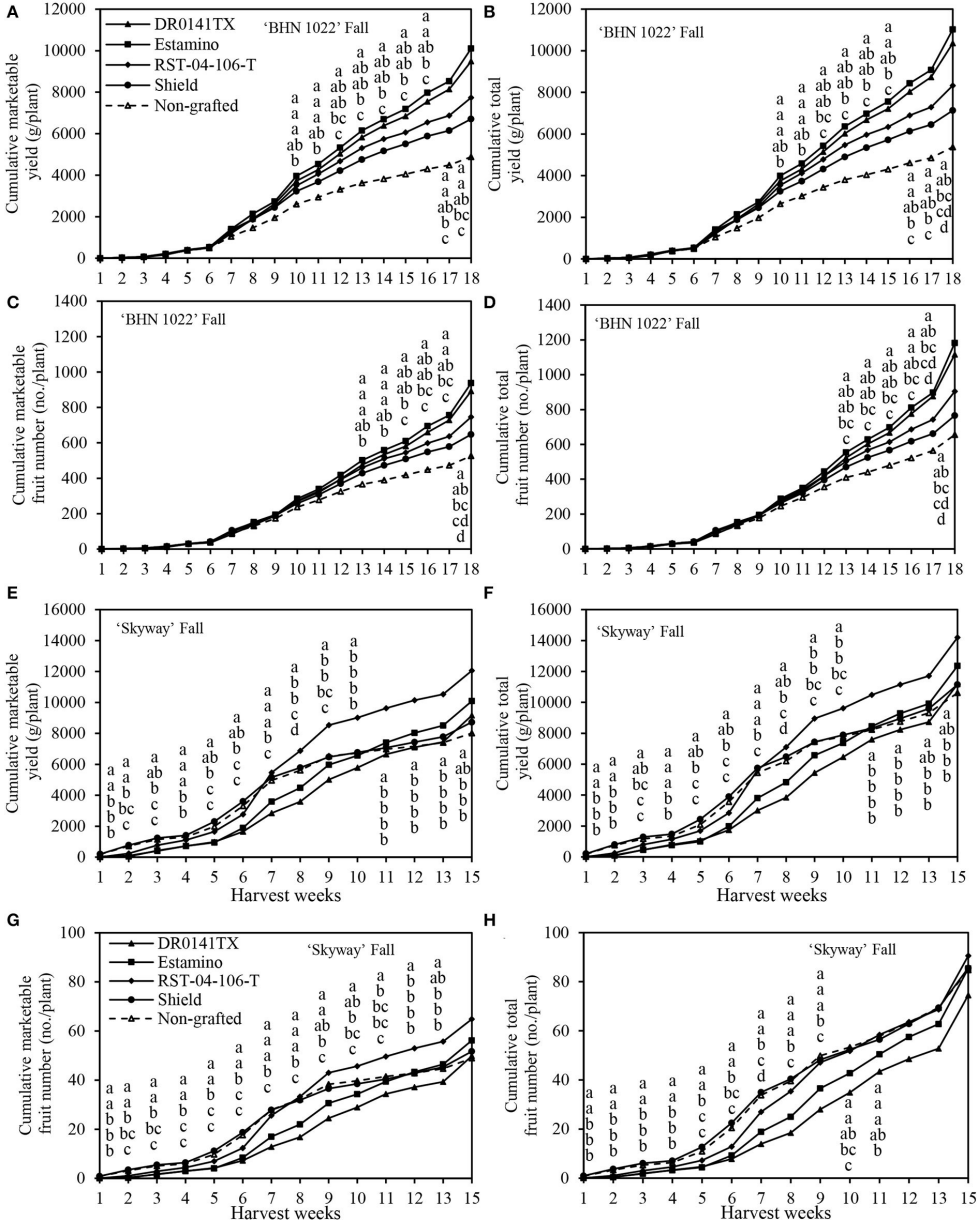
Cumulative marketable and total fruit weights and numbers of the grafted ‘BHN 1022' grape tomato and ‘Skyway' beefsteak tomato scions in the fall planting (24 September 2020 to 15 April 2021). Green fruits at final harvest were excluded. **(A)** Cumulative marketable yield of ‘BHN 1022'. **(B)** Cumulative total yield of ‘BHN 1022'. **(C)** Cumulative marketable fruit number of ‘BHN 1022'. **(D)** Cumulative total fruit number of ‘BHN 1022'. **(E)** Cumulative marketable yield of ‘Skyway'. **(F)** Cumulative total yield of ‘Skyway'. **(G)** Cumulative marketable fruit number of ‘Skyway'. **(H)** Cumulative total fruit number of ‘Skyway'. For the same harvest week, data with the same letter are not significantly different at *P* ≤ 0.05 according to Fisher's LSD test.

The weekly cumulative yield curves were also examined to help understand fruit yield development dynamics during the harvest season (with green fruits excluded at final harvest). Because of leaf mold in the 2020 spring, the harvest period lasted for only 8 weeks (Figure 3). In the fall planting, however, the harvest of ‘BHN 1022' grape tomatoes and ‘Skyway' beefsteak tomatoes continued for 18 and 15 weeks, respectively (Figure 4).

Rootstock impacts on yield changed over time and were reflected in temporal profiles for the different rootstock-scion combinations. For the ‘BHN 1022' grape-tomato scion, spring, and fall patterns were similar throughout most of the seasons for marketable and total cumulative yields and fruit numbers (Figures 3A–D, 4A–D). In the spring planting, the parameters increased slowly from harvest weeks (HWs) 1 to 2, rose quickly from 3 to 6 HWs, and then slowed from 6 HW to the final harvest (Figures 3A–D). No differences among the treatments were detected in the first 4 HWs. Values were greater when grafted onto ‘DR0141TX' or ‘Estamino' rootstock compared with those grafted onto the least-vigorous ‘Shield' or the non-grafted control beginning at 5HW for marketable and total cumulative yields, and at 6 HW for marketable and total cumulative fruit numbers. Grafting with ‘RST-04-106-T' resulted in greater marketable and total cumulative fruit yields and numbers than the non-grafted control starting from 6 HW, and it did not differ from plants grafted onto ‘Estamino' across all HW. In the fall planting (Figures 4A–D), the period when cumulative yield and fruit number increased slowly lasted until 6 HW, which was much longer compared with the spring planting. From 7 to 16 HWs, the cumulative yields of all the treatments appeared to increase almost linearly with different slopes. ‘Estamino'-grafted plants were higher than the non-grafted control in cumulative marketable and total yields from 10 HW and cumulative marketable and total fruit numbers from 13 HW until the end of harvest. ‘DR0141TX' and ‘RST-04-106-T' did not differ from ‘Estamino' across all the HWs except for 17 and 18 HWs for ‘RST-04-106-T'. ‘Shield' produced greater cumulative marketable and total yields than the non-grafted control during from 13 to 17 HWs but only had a greater cumulative marketable fruit number than the non-grafted control at 15 HW. The much greater increases in cumulative yields and fruit numbers at the end of the harvest period in the fall planting were not observed in the spring planting.

For the ‘Skyway' beefsteak tomato scion, rootstock effects on cumulative yields and fruit numbers varied greatly with planting seasons. In the spring planting, marketable and total cumulative yields and fruit numbers increased slowly for the first 2 HWs and then rose rapidly from 3 to 5 HWs (Figures 3E–H). From 6 to 8 HWs, cumulative yields increased at a slower rate than during the previous weeks in general. Differences among the treatments for cumulative marketable yield and fruit number were only observed at 2 and 3 HWs. At 2 HW, the non-grafted control had greater cumulative marketable yield than all the treatments except for ‘Estamino', while both ‘Estamino' and ‘Shield' resulted in similar cumulative marketable fruit numbers compared with the non-grafted control. At 2 HW, ‘Estamino' also led to higher cumulative marketable fruit yield and number than ‘DR0141TX'. Moreover, at 2 HW, all the treatments produced greater cumulative total yields than ‘DR0141TX' except ‘Shield'. At 3 HW, all the treatments had greater cumulative marketable fruit yields and numbers than ‘DR0141TX' except that ‘Shield' was similar to ‘DR0141TX' in cumulative marketable yield. At 7 HW, plants grafted onto ‘Estamino' or ‘DR0141TX' produced higher cumulative total yield than the non-grafted control, which was similar to plants grafted onto ‘RST-04-106-T' and ‘Shield'. At 8 HW, ‘Estamino'- or ‘DR0141TX'-grafted plants produced greatest cumulative total yields among all the treatments.

In the fall planting, cumulative marketable and total yields and fruit numbers remained low during the first 5 HWs for all the grafted plants (Figures 4E–H) and then increased fast until the end of harvest. Plants grafted onto ‘DR0141TX' or ‘Estamino' were lower in cumulative marketable and total yields and fruit numbers than the those grafted with ‘Shield' and the non-grafted control from 1 to 8 HWs. However, from 11 HW to the end of harvest, these four yield components of ‘Estamino'-grafted plants were similar to those grafted with ‘Shield' and the non-grafted control. ‘DR0141TX'-grafted plants had similar cumulative marketable and total yields to ‘Shield' and the non-grafted control from 11 HW to the end of harvest. Furthermore, plants grafted with ‘RST-04-106-T' produced similar cumulative marketable and total yields to that of the non-grafted control from 3 to 8 HWs. Interestingly, after 11 HWs, this treatment produced greater cumulative marketable yield and fruit number as well as cumulative total yield than those grafted with ‘Shield' and the non-grafted control. Grafting with ‘RST-04-106-T' also led to greater cumulative marketable and total yields and fruit numbers than that of ‘Estamino' and ‘DR01141TX' during 5–11 HW, except that for 6, 10, and 11 HWs, ‘RST-04-106-T' was similar to ‘Estamino' in cumulative total fruit number. ‘Shield' was similar to the non-grafted control in cumulative marketable yield and fruit number across all the HWs.

### Fruit cluster counts of ‘BHN 1022' in fall planting

Inflorescences, flower clusters, and fruit clusters of the ‘BHN 1022' grape tomato were counted at 55 (8 WAT), 103 (15 WAT), and 188 DAT (27 WAT), corresponding to 1 week before the first harvest, onset of the peak harvest period, and crop termination (Figures 2G–I). The ‘DR0141TX', ‘RST-04-106-T', and ‘Shield' rootstocks led to 58.5% more inflorescences than the non-grafted control at 55 DAT, whereas ‘Estamino'-grafted plants did not differ from the non-grafted control (Figure 2G). At 103 DAT, plants grafted onto the vigorous rootstocks (‘DR0141TX' and ‘Estamino') produced 184.2% more inflorescences than the non-grafted control, which was similar to the other two rootstock treatments.

The number of flower clusters on ‘BHN 1022' plants grafted with ‘DR0141TX' rootstocks was 45.8% higher than the non-grafted control at 55 DAT, while the other rootstock treatments were similar to the control (Figure 2H). At both 103 and 187 DAT, ‘DR0141TX' and ‘Estamino' led to greater flower cluster numbers compared with the non-grafted control. Moreover, The ‘BHN 1022' grafted onto ‘DR0141TX' and ‘Estamino' had more flower clusters than plants grafted with ‘RST-04-106-T' and ‘Shield' at 103 DAT, while the ‘DR0141TX' also resulted in a greater number of flower clusters than the two less vigorous rootstocks at 187 DAT. ‘RST-04-106-T' and ‘Shield' did not exhibit any significant impact on the flower cluster number of ‘BHN 1022'.

With respect to fruit clusters, no treatment differences were detected at 55 DAT (Figure 2I). However, at 187 DAT, plants grafted onto the vigorous ‘DR0141TX' or ‘Estamino' rootstock had 89.8% more fruit clusters than the non-grafted control, while ‘RST-04-106-T' and ‘Shield' did not show any significant effects.

### Vegetative, fruit and plant biomass, and harvest index

In both spring and fall plantings, plant biomass and vegetative biomass were affected by the rootstocks and scions but not their interactions (Tables 4, 5). For both scions in both planting seasons, the vigorous ‘DR0141TX' and ‘Estamino' rootstocks led to greater plant biomass and vegetative biomass than did ‘RST-04-106-T' and ‘Shield' rootstocks and the non-grafted controls except that plants grafted with ‘Estamino' and ‘RST-04-106-T' did not differ significantly in the fall planting (Tables 4, 5). Grafting onto ‘RST-04-106-T' increased plant biomass and vegetative biomass relative to ‘Shield' and the non-grafted controls in the fall planting, but such an effect was lacking in the spring planting. The plant biomass of ‘Skyway' was greater than that of ‘BHN 1022' in the spring planting, but no difference was found in the fall planting (Tables 4, 5). Moreover, ‘Skyway' consistently produced more vegetative biomass than ‘BHN 1022' in both planting seasons.

**Table 4:** Plant, vegetative, and fruit biomass, and harvest index (ratio between fruit biomass and plant biomass) of the grafted ‘BHN 1022' grape tomato and ‘Skyway' beefsteak tomato scions over the whole production season in the spring planting (14 February to 18 June 2020).

**Treatment**	**Plant biomass (g/plant DW)**	**Vegetative biomass (g/plant DW)**	**Fruit biomass (g/plant DW)**	**Harvest index**	
				**BHN 1022**	**Skyway**
**Rootstock (Rs)**					
DR0141TX	649.0 ± 37.3a	256.0 ± 15.0a	392.8 ± 24.0a	0.60 ± 0.01Ab	0.61 ± 0.01Ac
Estamino	568.3 ± 37.3b	214.6 ± 15.0b	353.8 ± 24.0b	0.60 ± 0.01Bb	0.64 ± 0.01Ab
RST-04-106-T	474.0 ± 37.3c	164.3 ± 15.0c	310.0 ± 24.0c	0.64 ± 0.01Aa	0.66 ± 0.01Aab
Shield	422.8 ± 37.3c	143.9 ± 15.0c	278.6 ± 24.0c	0.65 ± 0.01Aa	0.67 ± 0.01Aab
Non-grafted	436.0 ± 37.3c	157.1 ± 15.0c	278.9 ± 24.0c	0.60 ± 0.01Bb	0.68 ± 0.01Aa
**Scion (Sc)**					
BHN 1022	473.3 ± 34.6b	181.8 ± 13.3	291.4 ± 22.2b	–	
Skyway	546.8 ± 34.6a	192.6 ± 13.3	354.2 ± 22.2a	–	
***P*** **-Value**					
Rs	<0.001	<0.001	<0.001	<0.001	
Sc	<0.001	0.187	<0.001	0.035	
Rs × Sc	0.195	0.095	0.277	0.049	

DR0141TX, tomato scions grafted onto rootstock ‘DR0141TX'; Estamino, tomato scions grafted onto rootstock ‘Estamino'; RST-04-106-T, tomato scions grafted onto rootstock ‘RST-04-106-T'; Shield, tomato scions grafted onto ‘Shield'; Non-grafted, non-grafted tomato scion controls.

Mean ± SE (standard error); means followed by the same uppercase letter within a row and means followed by the same lowercase letter within a column are not significantly different at P ≤ 0.05 according to Fisher's LSD test.

**Table 5:** Plant, vegetative, and fruit biomass, and harvest index (ratio between fruit biomass and plant biomass) of the grafted ‘BHN 1022' grape tomato and ‘Skyway' beefsteak tomato scions over the whole production season in the fall planting (24 September 2020 to 15 April 2021).

**Treatment**	**Plant biomass (g/plant DW)**	**Vegetative biomass (g/plant DW)**	**Fruit biomass (g/plant DW)**		**Harvest index**
			**BHN 1022**	**Skyway**	
**Rootstock (Rs)**					
DR0141TX	1,731.3 ± 155.6a	895.9 ± 80.9a	952.3 ± 98.4Aa	718.5 ± 98.4Ba	0.49 ± 0.02c
Estamino	1,760.3 ± 155.6a	827.0 ± 80.9ab	1,018.3 ± 98.4Aa	848.3 ± 98.4Aa	0.53 ± 0.02bc
RST-04-106-T	1,475.9 ± 159.7b	677.7 ± 84.4b	733.3 ± 98.4Ab	845.6 ± 108.8Aa	0.56 ± 0.02b
Shield	1,061.3 ± 155.6c	378.3 ± 80.9c	700.0 ± 98.4Abc	666.0 ± 98.4Aa	0.65 ± 0.02a
Non-grafted	1,011.3 ± 159.7c	389.2 ± 84.4c	516.5 ± 98.4Ac	710.2 ± 108.8Aa	0.62 ± 0.02a
**Scion (Sc)**					
BHN 1022	1,262.6 ± 150.2	478.6 ± 75.8b	–		0.63 ± 0.01a
Skyway	1,553.4 ± 152.0	788.7 ± 77.4a	–		0.51 ± 0.01b
***P*** **-Value**					
Rs	<0.001	<0.001	0.002		<0.001
Sc	0.086	0.023	0.662		<0.001
Rs × Sc	0.262	0.180	0.037		0.082

DR0141TX, tomato scions grafted onto rootstock ‘DR0141TX'; Estamino, tomato scions grafted onto rootstock ‘Estamino'; RST-04-106-T, tomato scions grafted onto rootstock ‘RST-04-106-T'; Shield, tomato scions grafted onto ‘Shield'; Non-grafted, non-grafted tomato scion controls.

Mean ± SE (standard error); means followed by the same uppercase letter within a row and means followed by the same lowercase letter within a column are not significantly different at P ≤ 0.05 according to Fisher's LSD test.

In the spring planting, rootstock impacts on fruit biomass were similar to those on plant biomass and vegetative biomass (Table 4). In the fall planting, fruit biomass was affected by rootstock-scion interactions. The ‘BHN 1022' grafted onto ‘DR0141TX', ‘Estamino', or ‘RST-04-106-T' produced an average of 74.5% more fruit biomass than the non-grafted control (Table 5), whereas ‘Shield' was similar to the non-grafted control and ‘RST-04-106-T'. For ‘Skyway', fruit biomass was similar among all the treatments. Only when grafted onto ‘DR0141TX' did ‘BHN 1022' produce more fruit biomass than ‘Skyway'.

The harvest index was affected by the rootstocks and scions in both seasons, while the rootstock × scion interaction was also significant in the spring planting (Tables 4, 5). In the spring planting, the ‘RST-04-106-T' and ‘Shield' rootstocks increased the harvest indices of the ‘BHN 1022' grape tomato relative to the non-grafted control and the ‘DR0141TX' and ‘Estamino' treatments (Table 4). Grafting with ‘DR0141TX' or ‘Estamino' had negative or neutral effects on harvest index for both scions in both seasons, while grafting onto ‘Shield' led to greater (for ‘BHN 1022' in spring planting) or comparable harvest index to the non-grafted controls. ‘RST-04-106-T' effects on harvest index varied with scion and planting season. In the spring planting, ‘Skyway' had greater harvest index than ‘BHN 1022' when ungrafted or when grafted onto ‘Estamino'. In the fall planting, ‘BHN 1022' had higher harvest index than ‘Skyway'.

The Pearson correlation analysis of biomass and total fruit yield demonstrated differences in terms of the type and significance of relations among different measurements (Table 6). At *P* ≤ 0.05, the relationship between two parameters in the correlation analysis were interpreted as follows: correlation coefficient r between 0.9 and 1 or −0.9 to −1 (0.9 ≤ r ≤ 1 or −1 ≤ r ≤ −0.9) indicates a very strong relationship, 0.7 ≤ r <0.9 (−0.9 <r ≤ −0.7) indicates a strong relationship, 0.5 ≤ r <0.7 (−0.7 <r ≤ −0.5) indicates a moderate relationship, 0.3 ≤ r <0.5 (–.5 <r ≤ 0.3) indicates a weak relationship, and 0 ≤ r <0.3 (−0.3 <r ≤ 0) indicates a very weak or negligible relationship. In both planting seasons, fruit biomass was positively correlated with vegetative biomass, plant biomass, and total fruit yield, with the correlations being higher in the spring planting than in the fall planting (Table 6). In addition, vegetative biomass was very highly positively correlated with plant biomass during both planting seasons but was negatively correlated with harvest index. These correlations were moderate in the spring planting but high in the fall planting. Vegetative biomass showed a moderate to highly positive correlation with total fruit yield. A correlation between harvest index and total fruit yield was not detected in the spring planting. However, a low but significant negative correlation was detected between them in the fall planting.

**Table 6:** Correlation coefficient values based on Pearson correlation analysis among fruit biomass, vegetative biomass, plant biomass, harvest index, and total fruit yield in the spring planting (14 February to 18 June 2020) and the fall planting (24 September 2020 to 15 April 2021).

**Parameter**	**Fruit biomass**	**Vegetative biomass**	**Plant biomass**	**Harvest index**	**Total fruit yield**
**Spring planting**					
Fruit biomass					
Vegetative biomass	0.811***				
Plant biomass	0.966***	0.935***			
Harvest index	−0.031^NS^	−0.599***	−0.285^P = 0.074^		
Total fruit yield	0.911***	0.659***	0.843***	0.124^NS^	
**Fall planting**					
Fruit biomass					
Vegetative biomass	0.475**				
Plant biomass	0.794***	0.912***			
Harvest index	0.002^NS^	−0.858***	−0.591***		
Total fruit yield	0.736***	0.768***	0.874***	−0.474**	

NS, ^*^, ^**^, ^***^Non-significant or significant at P ≤ 0.05, 0.01, or 0.001, respectively, based on Pearson correlation analysis.

### Mineral nutrient contents in tomato fruit during peak harvest

Fruit N, P, K, Mg, Ca, and S contents on a dry weight basis were affected by the rootstocks and scions in both planting seasons (Tables 7, 8). In both planting seasons, fruit P, K, and Ca contents were increased by grafting with ‘DR0141TX' or ‘Estamino' in comparison with the non-grafted controls and those grafted onto ‘Shield'. The two rootstocks also resulted in positive or similar fruit N, Mg, and S contents as the non-grafted controls in both planting seasons. Grafting with ‘RST-04-106-T' decreased fruit Mg content in the spring planting but increased fruit P and Ca contents in the fall planting relative to the non-grafted controls (Tables 7, 8). Scion effects on fruit macronutrient content varied in planting seasons, except that the large-fruited ‘Skyway' consistently had greater fruit K than the small-fruited ‘BHN 1022' (Tables 7, 8). In the spring planting, ‘BHN 1022' had higher Mg, Ca, and S but lower K contents than ‘Skyway' (Table 7). In the fall planting, however, ‘BHN 1022' had lower N, P, K, Mg, and S contents than ‘Skyway' (Table 8).

**Table 7:** Fruit macronutrient content on a dry weight basis in tomatoes as affected by rootstock and scion cultivars in the spring planting (14 February to 18 June 2020).

**Treatment**	**N (mg/g DW)**	**P (mg/g DW)**	**K (mg/g DW)**	**Mg (mg/g DW)**	**Ca (mg/g DW)**	**S (mg/g DW)**
**Rootstock (Rs)**						
DR0141TX	21.20 ± 0.43	4.09 ± 0.27a	36.90 ± 0.77a	1.69 ± 0.03a	1.65 ± 0.08a	1.75 ± 0.03a
Estamino	21.03 ± 0.54	3.85 ± 0.26a	36.42 ± 0.89a	1.76 ± 0.04a	1.76 ± 0.09a	1.71 ± 0.04ab
RST-04-106-T	20.34 ± 0.43	3.46 ± 0.25b	32.88 ± 0.77b	1.50 ± 0.03c	1.38 ± 0.08b	1.63 ± 0.03c
Shield	20.34 ± 0.43	3.42 ± 0.24b	32.48 ± 0.77b	1.59 ± 0.03b	1.29 ± 0.08b	1.60 ± 0.03c
Non-grafted	19.66 ± 0.43	3.51 ± 0.25b	33.28 ± 0.77b	1.60 ± 0.03b	1.36 ± 0.08b	1.65 ± 0.03bc
**Scion (Sc)**						
BHN 1022	20.50 ± 0.30	3.79 ± 0.26	31.48 ± 0.84b	1.69 ± 0.03a	1.82 ± 0.06a	1.83 ± 0.03a
Skyway	20.53 ± 0.33	3.54 ± 0.26	37.30 ± 0.86a	1.57 ± 0.03b	1.16 ± 0.06b	1.51 ± 0.03b
***P*** **-Value**						
Rs	0.122	<0.001	<0.001	<0.001	<0.001	0.002
Sc	0.952	0.302	0.003	0.029	0.001	<0.001
Rs × Sc	0.218	0.921	0.806	0.197	0.173	0.529

Ripe ‘BHN 1022' grape tomatoes were harvested at 90 days after transplanting (DAT). Breaker-stage fruits of ‘Skyway' beefsteak tomatoes were harvested at 97 DAT and then allowed to ripen at 20°C until fully red.

DR0141TX, tomato scions grafted onto rootstock ‘DR0141TX'; Estamino, tomato scions grafted onto rootstock ‘Estamino'; RST-04-106-T, tomato scions grafted onto rootstock ‘RST-04-106-T'; Shield, tomato scions grafted onto ‘Shield'; Non-grafted, non-grafted tomato scion controls.

Mean ± SE (standard error); means within a column followed by the same letter are not significantly different at P ≤ 0.05 according to Fisher's LSD test.

**Table 8:** Fruit macronutrient content on a dry weight basis in grafted tomatoes as affected by rootstock and scion cultivars in the fall planting (24 September 2020 to 15 April 2021).

**Treatment**	**N (mg/g DW)**	**P (mg/g DW)**	**K (mg/g DW)**	**Mg (mg/g DW)**	**Ca (mg/g DW)**	**S (mg/g DW)**
**Rootstock (Rs)**						
DR0141TX	24.11 ± 0.82a	4.56 ± 0.17a	36.41 ± 0.96a	1.49 ± 0.05	2.60 ± 0.13ab	2.14 ± 0.06
Estamino	23.09 ± 0.80ab	4.47 ± 0.17a	36.13 ± 0.96a	1.53 ± 0.05	2.85 ± 0.13a	2.11 ± 0.06
RST-04-106-T	22.44 ± 0.86ab	4.36 ± 0.18a	35.71 ± 1.05ab	1.56 ± 0.05	2.44 ± 0.14bc	1.98 ± 0.06
Shield	20.22 ± 0.75c	3.67 ± 0.15b	32.98 ± 0.96b	1.53 ± 0.05	2.26 ± 0.13cd	2.13 ± 0.06
Non-grafted	21.58 ± 0.78bc	3.66 ± 0.16b	33.01 ± 0.96b	1.51 ± 0.05	2.10 ± 0.13d	2.01 ± 0.06
**Scion (Sc)**						
BHN 1022	20.60 ± 0.60b	3.83 ± 0.10b	30.61 ± 0.70b	1.42 ± 0.04b	2.29 ± 0.12	1.98 ± 0.04b
Skyway	24.01 ± 0.66a	4.45 ± 0.11a	39.09 ± 0.72a	1.63 ± 0.04a	2.62 ± 0.12	2.17 ± 0.04a
***P*** **-Value**						
Rs	0.013	<0.001	0.025	0.857	<0.001	0.277
Sc	0.003	<0.001	<0.001	0.005	0.096	0.002
Rs × Sc	0.776	0.403	0.921	0.936	0.419	0.684

Ripe ‘BHN 1022' grape tomatoes were harvested at 106 days after transplanting (DAT). Breaker-stage fruits of ‘Skyway' beefsteak tomatoes were harvested at 131 DAT and then allowed to ripen at 20°C until fully red.

DR0141TX, tomato scions grafted onto rootstock ‘DR0141TX'; Estamino, tomato scions grafted onto rootstock ‘Estamino'; RST-04-106-T, tomato scions grafted onto rootstock ‘RST-04-106-T'; Shield, tomato scions grafted onto ‘Shield'; Non-grafted, non-grafted tomato scion controls.

Mean ± SE (standard error); means within a column followed by the same letter are not significantly different at P ≤ 0.05 according to Fisher's LSD test.

Fruit Zn and Fe contents were also affected by the rootstocks in both planting seasons, with generally higher levels observed in the ‘DR0141TX' and ‘Estamino' treatments relative to the non-grafted controls and those grafted onto ‘RST-04-106-T' or ‘Shield' (Tables 9, 10). The rootstock × scion interaction was observed for fruit Mn content in the spring planting and for fruit B content in the fall planting, in addition to the rootstock effect on fruit B in the spring planting and fruit Mn in the fall planting. Grafting with ‘RST-04-106-T' reduced fruit B contents for both scions in the spring planting, whereas it increased the fruit B content for ‘Skyway' in the fall planting. ‘DR0141TX' and ‘Estamino' resulted in higher Mn contents for both scions in the fall planting, but such effects were only observed for ‘BHN 1022' in the spring planting. ‘DR0141TX' also increased fruit Cu content in the fall planting. Compared with ‘BHN 1022', ‘Skyway' fruits demonstrated higher B but lower Cu contents in the spring planting, with higher Mn and Cu contents in the fall planting.

**Table 9:** Fruit micronutrient content on a dry weight basis in grafted tomatoes as affected by rootstock and scion cultivars in the spring planting (14 February to 18 June 2020).

**Treatment**	**B (μg/g DW)**	**Zn (μg/g DW)**	**Mn (**μ**g/g DW)**		**Fe (μg/g DW)**	**Cu (μg/g DW)**
			**BHN 1022**	**Skyway**		
**Rootstock (Rs)**						
DR0141TX	10.00 ± 0.31a	22.63 ± 0.93a	13.75 ± 0.96Aa	10.00 ± 0.96Ba	47.25 ± 2.08a	13.88 ± 1.16
Estamino	10.32 ± 0.34a	21.56 ± 1.08ab	14.25 ± 0.96Aa	12.08 ± 1.32Aa	49.44 ± 2.48a	13.64 ± 1.38
RST-04-106-T	9.25 ± 0.31b	19.88 ± 0.93bc	9.75 ± 0.96Ab	9.25 ± 0.96Aa	39.63 ± 2.08bc	13.25 ± 1.16
Shield	9.88 ± 0.31a	17.50 ± 0.93d	10.00 ± 0.96Ab	10.50 ± 0.96Aa	36.75 ± 2.08c	14.38 ± 1.16
Non-grafted	10.13 ± 0.31a	18.63 ± 0.93cd	10.25 ± 0.96Ab	11.75 ± 0.96Aa	41.88 ± 2.08b	13.75 ± 1.16
**Scion (Sc)**						
BHN 1022	9.25 ± 0.38b	20.80 ± 0.86	–		43.65 ± 1.68	17.70 ± 1.05a
Skyway	10.58 ± 0.39a	19.27 ± 0.89	–		42.33 ± 1.76	9.86 ± 1.09b
***P*** **-Value**						
Rs	0.006	<0.001	0.007		<0.001	0.943
Sc	0.050	0.230	0.135		0.511	0.011
Rs × Sc	0.056	0.862	0.043		0.064	0.230

Ripe ‘BHN 1022' grape tomatoes were harvested at 90 days after transplanting (DAT). Breaker-stage fruits of ‘Skyway' beefsteak tomatoes were harvested at 97 DAT and then allowed to ripen at 20°C until fully red.

DR0141TX, tomato scions grafted onto rootstock ‘DR0141TX'; Estamino, tomato scions grafted onto rootstock ‘Estamino'; RST-04-106-T, tomato scions grafted onto rootstock ‘RST-04-106-T'; Shield, tomato scions grafted onto ‘Shield'; Non-grafted, non-grafted tomato scion controls.

Mean ± SE (standard error); means followed by the same uppercase letter within a row and means followed by the same lowercase letter within a column are not significantly different at P ≤ 0.05 according to Fisher's LSD test.

**Table 10:** Fruit micronutrient content on a dry weight basis in grafted tomatoes as affected by rootstock and scion cultivars in the fall planting (24 September 2020 to 15 April 2021).

**Treatment**	**B (**μ**g/g DW)**		**Zn (μg/g DW)**	**Mn (μg/g DW)**	**Fe (μg/g DW)**	**Cu (μg/g DW)**
	**BHN 1022**	**Skyway**				
**Rootstock (Rs)**						
DR0141TX	10.25 ± 0.59Aa	8.75 ± 0.59Ab	23.63 ± 0.68a	25.25 ± 1.47a	51.25 ± 2.08b	7.38 ± 0.41a
Estamino	9.50 ± 0.59Aa	10.00 ± 0.59Aab	24.00 ± 0.68a	24.25 ± 1.47a	57.50 ± 2.08a	5.50 ± 0.41b
RST-04-106-T	8.75 ± 0.59Ba	11.32 ± 0.69Aa	20.10 ± 0.74b	22.38 ± 1.56a	44.19 ± 2.25c	5.33 ± 0.45b
Shield	9.50 ± 0.59Aa	9.25 ± 0.59Ab	19.13 ± 0.68b	16.88 ± 1.47b	39.38 ± 2.08c	5.63 ± 0.41b
Non-grafted	9.25 ± 0.59Aa	9.25 ± 0.59Ab	18.75 ± 0.68b	18.50 ± 1.47b	39.63 ± 2.08c	5.63 ± 0.41b
**Scion (Sc)**						
BHN 1022			21.05 ± 0.50	17.75 ± 1.29b	44.50 ± 1.57	4.40 ± 0.26b
Skyway			21.19 ± 0.51	25.15 ± 1.31a	48.28 ± 1.61	7.38 ± 0.27a
***P*** **-Value**						
Rs	0.718		<0.001	<0.001	<0.001	0.010
Sc	0.644		0.822	0.017	0.142	<0.001
Rs × Sc	0.025		0.103	0.987	0.156	0.633

Ripe ‘BHN 1022' grape tomatoes were harvested at 106 days after transplanting (DAT). Breaker-stage fruits of ‘Skyway' beefsteak tomatoes were harvested at 131 DAT and then allowed to ripen at 20°C until fully red.

DR0141TX, tomato scions grafted onto rootstock ‘DR0141TX'; Estamino, tomato scions grafted onto rootstock ‘Estamino'; RST-04-106-T, tomato scions grafted onto rootstock ‘RST-04-106-T'; Shield, tomato scions grafted onto ‘Shield'; Non-grafted, non-grafted tomato scion controls.

Mean ± SE (standard error); means followed by the same uppercase letter within a row and means followed by the same lowercase letter within a column are not significantly different at P ≤ 0.05 according to Fisher's LSD test.

### Root-knot nematode galling index ratings

For both planting seasons, the ‘Skyway' treatment had similar root-knot nematode galling index ratings ranging from 0 to 0.8 (Table 11). In the spring planting, the non-grafted ‘BHN 1022' had a galling index rating of 1.6 vs. 0 for all the grafted ‘BHN 1022' treatments. In the fall planting, the galling rating of the non-grafted ‘BHN 1022' was 4.1, in contrast to the scores near 0 for all the grafted treatments.

**Table 11:** Nematode galling index ratings (0–10 rating) of grafted beefsteak (‘Skyway') and grape (‘BHN 1022') tomatoes at plant termination as affected by rootstock and scion cultivars.

**Rootstock**	**Spring planting**		**Fall planting**	
	**BHN 1022**	**Skyway**	**BHN 1022**	**Skyway**
DR0141TX	0.0 ± 0.0b	0.0 ± 0.0	0.2 ± 0.1b	0.1 ± 0.1
Estamino	0.0 ± 0.0b	0.0 ± 0.0	0.0 ± 0.0b	0.0 ± 0.0
RST-04-106-T	0.0 ± 0.0b	0.0 ± 0.0	0.0 ± 0.0b	0.0 ± 0.0
Shield	0.0 ± 0.0b	0.0 ± 0.0	0.2 ± 0.2b	0.1 ± 0.1
Non-grafted	1.6 ± 0.8a	0.1 ± 0.1	4.1 ± 0.8a	0.8 ± 0.6
*P*-Value	0.006	0.406	0.006	0.330

Root-knot nematode galling index proposed by Zeck (1971).

DR0141TX, tomato scions grafted onto rootstock ‘DR0141TX'; Estamino, tomato scions grafted onto rootstock ‘Estamino'; RST-04-106-T, tomato scions grafted onto rootstock ‘RST-04-106-T'; Shield, tomato scions grafted onto ‘Shield'; Non-grafted, non-grafted tomato scion controls.

Mean ± SE (standard error); means within a column followed by the same letter are not significantly different at P ≤ 0.05 according to Wilcoxon Each Pair test.

## Discussion

### Tomato yield components

Compared with the intraspecific rootstock ‘Shield', the interspecific rootstock ‘Estamino' consistently increased both marketable and total yields while its effects on fruit numbers varied with scion cultivar. These results are generally in line with the greenhouse study by Leonardi and Giuffrida (2006) who found that grafting onto an interspecific rootstock (*S. lycopersicum* × *S. habrochaites*) increased the total fruit number and marketable yield of the tomato scion, whereas grafting onto intraspecific rootstocks had negligible effects. However, Buller et al. (2013) reported no increase in marketable yield when grafting tomato with interspecific rootstocks in the field with no verticillium wilt. According to Arthur et al. (2021), the interspecific rootstocks ‘Emperador' and ‘Maxifort' (*S. lycopersicum* × *S. habrochaites*) did not affect the total yield of six tomato scion cultivars of various sizes (14.3–386.8 g/fruit), while the rootstock effect on marketable yield varied with scion cultivars, which seemed to show no relation with fruit size. Contrasting results from different studies could have arisen from the different rootstock and scion combinations, production systems, growing conditions (presence/absence of stress), or combined factors.

In general, rootstock vigor was positively associated with fruit yield, while the least vigorous rootstock, ‘Shield', had little effect unless grafted with the small-fruited ‘BHN 1022' scion in the fall planting. The increase in fruit yield from grafting with vigorous rootstocks ‘DR0141TX' and ‘Estamino' could be partially driven by increased water content in fruit, as was manifested by lower dry matter content for both scions in both planting seasons (Gong, 2022). Mauro et al. (2020) also reported that the dry weight-based total fruit yields of cherry tomato plants grafted with four *S. lycopersicum* × *S. habrochaites* interspecific rootstocks was lower than those of the non-grafted control, while the fresh weight-based total fruit yields of grafted plants were greater than or similar to the non-grafted control. Studies by Ho et al. (1987) suggested that water accumulation plays a major role in determining final fruit size. When individual fruit dry biomass stays the same, greater water accumulation leads to greater fresh weight along with lower dry matter content. Furthermore, previous studies have demonstrated significantly positive relationships between yield characteristics of grafted tomato and total root length, root surface area, and root dry weight (Bayindir and Kandemir, 2022), and grafted tomato plants with growth improvement have been found to possess enhanced root length density in the upper 15 cm of soil (Djidonou et al., 2019). The more developed root system of vigorous rootstocks could help absorb and transport more water, potentially leading to higher water accumulation in fruit. More research is needed to examine the contributions of fruit dry biomass and water accumulation to overall fruit yield of tomatoes grafted with vigorous rootstocks and better characterize the role of the modified root system.

Positive effects of grafting with ‘RST-04-106-T' on fruit yields, especially in the fall planting (through the winter), were observed in our study, suggesting that ‘RST-04-106-T' might perform better in cold environments as no yield improvement was observed in previous studies when majority of the harvests occurred in warmer months (Kunwar et al., 2015; Lang and Nair, 2019). Suchoff et al. (2019) found that in the field with a history of bacterial wilt, ‘RST-04-106-T' increased tomato marketable yield compared with the non-grafted control, but yield improvement of grafted plants was not observed when bacterial wilt was absent. These results suggest that considering biotic and abiotic stress factors at a production site is critical for rootstock selection to benefit tomato productivity.

Although there appeared to be a lack of rootstock impact on fruit number per plant for the large-fruited beefsteak tomato ‘Skyway' during either season, reverse trends were observed for the ‘BHN 1022' grape tomato that produced more fruit on all grafted plants except those on the ‘Shield' rootstock. This finding highlights the scion-dependent outcome in grafted tomato production, which was also reported by Frey et al. (2020b). The yield improvement of the ‘BHN 1022' grafted with vigorous rootstocks could be partially ascribed to the greater number of fruit clusters produced throughout the season. The greater inflorescence and flower cluster numbers counted at 103 DAT (HW 6) for the ‘BHN 1022' grafted with the more vigorous rootstocks corresponded to greater weekly yields in the following weeks relative to the ‘BHN 1022' grafted with less vigorous rootstocks. In addition, at crop termination, ‘DR0141TX' resulted in more flower clusters than ‘RST-04-106-T', ‘Shield', and the non-grafted control, indicating its potential productivity if greater season extension could be achieved. Flower cluster numbers were similar between ‘DR0141TX' and ‘Estamino', suggesting comparable levels of potential productivity between vegetative and generative rootstocks.

It is noteworthy that in the fall planting the low yield of the non-grafted ‘BHN 1022' grape tomato was partially due in part to a relatively high nematode infestation (galling index > 4), while in the spring, the level of galling was less (index <2). As revealed by Bridge and Page (1980), root-knot nematode (RKN) galling ratings ≥4 (based on a 0–10 scale) may lead to significant yield losses. Although tomato was rotated with cowpea in the summer, the lack of nematode resistance by ‘BHN 1022' could have led to the high galling rating in the fall planting (Ozores-Hampton and McAvoy, 2017). The ‘Shield' rootstock has intermediate resistance to nematodes (https://www.rijkzwaanusa.com/find-your-variety/rootstock/shield-rz) and has a nematode galling index of 0 and 0.2 when grafted with ‘BHN 1022' grape tomatoes in the spring and fall plantings, respectively. This could be part of the reason that the ‘Shield'-grafted ‘BHN 1022' had a higher yield than the non-grafted ‘BHN 1022' in the fall planting but no effect in the spring planting. The ‘Skyway' scion, on its own, has intermediate resistance to root-knot nematodes (Ozores-Hampton and McAvoy, 2017); thus, it is not surprising to see similar nematode galling indices between the non-grafted ‘Skyway' and rootstock-grafted plants. It is also noteworthy that in the fall planting, blotchy ripening was observed mainly on the ‘Skyway' beefsteak tomato but not on the ‘BHN 1022' grape tomato fruits. This might have affected the extent of rootstock impacts on marketable yield, but the percentage of fruit with blotchy ripening did not exceed 10% out of the whole-season harvests.

The average marketable fruit weight of ‘BHN 1022' grape tomato scion was 9.6% less in the fall than in the spring, and a reduction of 45.3% was found for the large-fruited ‘Skyway' (Table 3). According to Adams et al. (2001), low temperatures reduce the absolute volumetric growth rates of tomato fruits and delay the time at which absolute growth rate became maximal. Fall temperatures may have had similar effects in the present study, thus contributing to lower fruit weight at harvest.

### Cumulative yield

Compared with the beefsteak tomato ‘Skyway', the grape tomato ‘BHN 1022' responded more markedly to the vigorous rootstocks for increased fruit production at mid and late harvests relative to the less vigorous rootstocks and the non-grafted control. Djidonou et al. (2017) found that rootstocks promoted beefsteak tomato marketable and total yield most strongly during the mid-harvest period in a greenhouse pot study, which is more in line with our findings with the grape tomato scion. In the present study, rootstock effects on early harvest differed between the scions. For ‘BHN 1022', grafting with the four selected rootstocks did not show any negative impacts on fruit production in early harvest for both planting seasons. However, lower yield at early harvest was observed when ‘Skyway' was grafted with certain rootstocks, especially in the fall planting. More research is warranted in terms of rootstock effects on fruit production dynamics.

Darawsheh and Bouranis (2006) reported that for small-fruited tomato, fruit set and maturation were delayed when the growing-season temperature averaged 14.4°C and ranged between 9.5 and 19.0°C, which could postpone fruit harvest. In the present study, although the average daily temperature in the fall planting season varied between 8.5 and 27.6°C, the first harvest of ‘BHN 1022' was not delayed as the harvest began at 10 WAT in both planting seasons. Moreover, the cold night temperature appeared to show a little negative influence on grape tomato fruit development as the weekly yield increased rapidly from HW 6 when the minimum air temperature dropped to 1.5°C and remained below 5°C through HW 10 (Figure 1B). However, there was a much longer period in the fall when cumulative yield increased more slowly than in the spring, which warrants further research. In contrast to the ‘BHN 1022' grape tomato, depressive effects of cold night temperatures were evident on the development of the beefsteak tomato ‘Skyway'. The first fall harvest was delayed to 14 WAT compared with 10 WAT in the spring. Furthermore, the weekly yield of ‘Skyway' did not increase significantly until HW 6, after which the minimal night temperature was above 2°C (except at 27 WAT). It seems that regardless of grafting status, the beefsteak tomato was generally more sensitive to cold, especially to the minimal night temperature, than was the grape tomato. This concurs with Riga (2015) who found that for beefsteak tomato, rootstock genotype did not mitigate the negative effects of low temperature and light conditions on fruit production. It has been suggested that different tomato fruit types may have different mechanisms for low night temperature tolerance, possibly due to different proline accumulation (Yang et al., 2021). More in-depth research is needed to elucidate the underlying physiological responses to low night temperature of tomatoes of different fruit sizes.

Very likely, fruit temperatures might have affected the fruit ripening process of the two types of tomato scions in the present study. We found that the mass average temperature, measured internally at a depth of 1/3 of the fruit radius (Smith and Bennett, 1965), changed almost instantly with fluctuations in ambient air temperatures for grape tomato fruits but was delayed ~1 h for either warming or cooling of beefsteak tomato fruits at air temperatures between 1.5 and 25°C (unpublished data). As a result, under the same environment, beefsteak tomatoes might have accumulated less heat than grape tomatoes within each 24-h period. In other words, the mass average temperature of beefsteak tomato fruit within each 24-h period was likely lower than that of grape tomato fruits. The lower fruit temperature of beefsteak fruits could lead to delay in fruit development and ripening as specific biological changes require an optimal temperature range. If temperatures are below a given minimum, key reactions either slow or do not begin. Previous work has shown that below 12°C, almost no growth is expected for tomato (Criddle et al., 1997). The lower heat accumulation/average fruit temperature of beefsteak tomato fruit compared with grape tomato fruit also implied that fruit size might be negatively associated with fruit heat accumulation. In the fall planting, the slower increase in the cumulative yield of ‘Skyway' grafted onto the two most vigorous rootstocks ‘DR0141TX' and ‘Estamino' compared with the least vigorous rootstock ‘Shield' examined in the present study and the non-grafted control might be related to relatively higher average fruit weight from grafted ‘DR0141TX' and ‘Estamino' plants, thus longer time would have been needed to reach the breaker stage under the low temperature encountered during winter. In addition, around twice as many green fruits were harvested from grafted ‘DR0141TX' and ‘Estamino' than from grafted ‘Shield' or the non-grafted control at crop termination in the fall planting, indicating that the relatively lower cumulative yields throughout the season of the former two treatments might not be due to lower fruit production capability but to the possibility that fewer fruits developed to harvest standard in the given environmental conditions.

### Plant biomass production and partitioning

In general, vigorous rootstocks showed positive impacts on grafted tomato whole-season vegetative, fruit, and plant biomass (except for grafting with the ‘Skyway' scion in fall planting) compared with those grafted with the least vigorous rootstock ‘Shield' and the non-grafted control. The impacts of medium vigorous rootstock ‘RST-04-106-T' on plant biomass production and partitioning differed between planting seasons. However, harvest index was decreased by the two vigorous rootstocks. Lang et al. (2020) also found that grafting a determinate beefsteak tomato onto ‘DR0141TX' or ‘Estamino' rootstocks resulted in greater aboveground biomass at the end of the cropping cycle compared with the non-grafted control and ‘RST-04-106-T'-grafted plant in two separate years, while the latter two did not differ from each other. This result concurs with our findings in the spring planting.

The interspecific rootstock ‘Estamino' led to greater plant, vegetative, and fruit biomass (on a dry weight basis) in contrast to the intraspecific rootstock ‘Shield' and the non-grafted controls, which could be due to higher light use efficiency (Higashide et al., 2014). Barrett et al. (2012) also found that for the large-fruited ‘Brandywine' tomato, grafting with an interspecific rootstock produced higher levels of plant biomass compared with an intraspecific rootstock treatment. Mauro et al. (2020) reported that in general, grafting cherry tomatoes with *S. lycopersicum* × *S. habrochaites* rootstocks increased plant biomass and vegetative biomass but decreased fruit biomass and harvest index relative to an intraspecific rootstock and the non-grafted control, while the latter two were similar. This suggested that certain *S. lycopersicum* × *S. habrochaites* rootstocks promoted overall plant growth, but that the improved vegetative growth came at the expense of fruit production. In the present study, similarities were also observed in balance between increases in plant and vegetative biomass, relative to decreases in harvest indices with grafting onto the vigorous ‘Estamino' vs. the less-vigorous, intraspecific rootstock (‘Shield') and the non-grafted control. However, the fruit biomass was also increased by grafting with ‘Estamino' for both scions in the spring, and for ‘BHN 1022' in the fall, suggesting that by enhancing vegetative growth, the rootstock might increase “source” size, thus potentially supporting greater fruit load. In addition, the negative correlation between vegetative biomass and harvest index had a moderate to high level of statistical significance, suggesting that the rootstocks disproportionally enhanced the vegetative growth. Kyriacou et al. (2017) pointed out that vigorous rootstocks may act as additional sinks for assimilates, thus limiting photosynthate availability for fruit production. When pooled over all rootstock-scion combinations, the harvest index in the spring planting was greater than that in the fall planting. The sink strength of tomato fruit has been suggested to increase with temperature (Ho and Hewitt, 1986); thus, the higher temperature in the spring planting may facilitate partitioning of photosynthates into fruits.

The present results did not support previous designations of “generative” rootstocks (‘Estamino') directing more resources to reproductive parts of the scion and “vegetative” rootstocks (‘DR0141TX') favoring leafy growth by scions. Here, both rootstocks had similar effects on the scions and planting seasons tested.

### Fruit minerals

Fruit from plants grafted with ‘DR0141TX' or ‘Estamino' had higher P, K, Ca, Zn, and Fe contents than the non-grafted controls for both scions in both planting seasons on a dry weight basis. This result was similar to our previous findings with two grape tomato scions grafted onto the same vigorous rootstocks under organic high tunnel production (Gong et al., 2022). The consistency of responses indicates that the two rootstocks probably have similar capacities to take up and transport these mineral elements. However, rootstock impacts on fruit N, Mg, S, B, Mn, Fe, and Cu varied with planting seasons. Sabatino et al. (2021) reported that rootstocks increased the fruit N, P, Ca, Mg, and S contents of cherry tomato relative to the non-grafted control in greenhouse conditions. As pointed out by Khah et al. (2006), rootstock effects on specific mineral elements in tomato fruits differed between production systems.

Different root morphology and architecture (Suchoff et al., 2017), profiles of transporters expressed (Albornoz et al., 2018), and rootstock genetics (Asins et al., 2017) could lead to contrasts in their capacity to access and take up different mineral nutrients. The synthesis of more efficient root plasma membrane transporters and a larger root volume could confer higher uptake efficiency by a given rootstock than by non-grafted scion control (Albornoz et al., 2018). Moreover, rootstocks also affected mineral uptake concentrations (nutrient-to-water uptake ratios), as Savvas et al. (2017) found that grafted tomato plants had higher N, P, Ca, Fe, Mn, and B uptake concentrations than the non-grafted control, suggesting that the sap flowing to fruit might contain higher concentrations of minerals. In the case of the ‘DR0141TX' and ‘Estamino'-grafted tomatoes in the present study, greater water transportation to fruits, as was manifested by lower dry matter content, might further contribute to the mineral transport to fruit.

In terms of fruit mineral content on an FW basis, in general, neutral or negative effects of rootstocks were observed (data not shown). This result is consistent with our previous findings for two grape tomato scions on the two vigorous rootstocks used here under organic production in high tunnels (Gong et al., 2022). The observed impact could be due to the lower dry matter content of rootstock-grafted fruit (data not shown), which was also observed by Turhan et al. (2011) and Djidonou et al. (2016).

## Conclusions

In this study, effects of diverse tomato rootstocks were examined on contrasting tomato scion types and during different growing seasons. Rootstock impacts were quantified for fruit yield, biomass production, and fruit mineral content from a small-fruited grape tomato scion (‘BHN 1022') and a large-fruited beefsteak tomato scion (‘Skyway'). The vigorous rootstocks (‘DR0141TX' and ‘Estamino') generally increased fruit yields for both scions during both growing seasons, except for the large-fruited ‘Skyway' grafted onto ‘DR0141TX' in the fall planting. The positive effects of ‘RST-04-106-T' on fruit yield varied with scion and planting season and were most manifested when grafted with ‘Skyway' in the fall planting. The least vigorous rootstock, ‘SHIELD RZ F1 (61-802)', led to yields similar to the non-grafted controls except when grafted with ‘BHN 1022' in the fall planting. Higher yields of grafted plants were mainly ascribed to greater fruit numbers. In the fall planting, cold temperatures delayed the first harvest of the ‘Skyway' beefsteak tomato and decreased the yield during the early harvest period. In contrast, the production of ‘BHN 1022' grape tomato was less affected. The two most vigorous rootstocks, ‘DR0141TX' and ‘Estamino', generally increased the plant vegetative and fruit biomass of both scions during both planting seasons, except for the large-fruited ‘Skyway' in the fall planting. The effects of the ‘RST-04-106-T' rootstock varied with planting season. Harvest index was moderately to highly negatively correlated with vegetative biomass. The ‘DR0141TX', ‘Estamino', and ‘RST-04-106-T' rootstocks had neutral or negative impacts on harvest index relative to the non-grafted controls except for the ‘RST-04-106-T' grafted with ‘BHN 1022' in the spring planting. The scions grafted onto ‘SHIELD RZ F1 (61-802)' had similar biomass and harvest index as the non-grafted controls except when grafted with ‘BHN 1022' in the spring planting. For fruit mineral content, the scions grafted with the vigorous ‘DR0141TX' and ‘Estamino' rootstocks had higher fruit P, K, Ca, Zn, and Fe contents on a dry weight basis during both seasons.

Tomato scion yield, biomass production, and fruit mineral contents varied with planting season and were affected by the rootstocks in a way that may not strictly follow rootstock vigor. In this study on determinate grape and beefsteak tomato production under organically managed high tunnel conditions in north central Florida, the “vegetative” and “generative” rootstocks showed similar impacts on tomato plant growth and development, fruit yield, and biomass partitioning. Future research with different production systems and management practices and contrasting scion genotypes is needed to better understand the impacts of rootstocks with different vigor and other characteristics on plant biomass production and its implications for fruit yield development.

## Data availability statement

The raw data supporting the conclusions of this article will be made available by the authors, without undue reservation.

## Author contributions

TG designed and performed the experiments, analyzed the data, and drafted the manuscript. XZ and JB supervised TG to design and conduct the experiments and reviewed and edited the manuscript. SH and KK offered advice on research information synthesis and helped revise the manuscript. All authors contributed to the article and approved the submitted version.
